# The Role of Clathrin in Post-Golgi Trafficking in *Toxoplasma gondii*

**DOI:** 10.1371/journal.pone.0077620

**Published:** 2013-10-11

**Authors:** Manuela S. Pieperhoff, Miriam Schmitt, David J. P. Ferguson, Markus Meissner

**Affiliations:** 1 Wellcome Trust Centre for Molecular Parasitology, Institute of Infection, Immunity & Inflammation, College of Medical, Veterinary and Life Sciences, University of Glasgow, Glasgow, United Kingdom; 2 Mammalian Cell Cycle Control Mechanisms, German Cancer Research Center, Heidelberg, Germany; 3 Nuffield Department of Clinical Laboratory Medicine, University of Oxford, Oxford, United Kingdom; Centre National de la Recherche Scientifique, France

## Abstract

Apicomplexan parasites are single eukaryotic cells with a highly polarised secretory system that contains unique secretory organelles (micronemes and rhoptries) that are required for host cell invasion. In contrast, the role of the endosomal system is poorly understood in these parasites. With many typical endocytic factors missing, we speculated that endocytosis depends exclusively on a clathrin-mediated mechanism. Intriguingly, in *Toxoplasma gondii* we were only able to observe the endogenous clathrin heavy chain 1 (CHC1) at the Golgi, but not at the parasite surface. For the functional characterisation of *Toxoplasma gondii* CHC1 we generated parasite mutants conditionally expressing the dominant negative clathrin Hub fragment and demonstrate that CHC1 is essential for vesicle formation at the trans-Golgi network. Consequently, the functional ablation of CHC1 results in Golgi aberrations, a block in the biogenesis of the unique secretory microneme and rhoptry organelles, and of the pellicle. However, we found no morphological evidence for clathrin mediating endocytosis in these parasites and speculate that they remodelled their vesicular trafficking system to adapt to an intracellular lifestyle.

## Introduction

Apicomplexan parasites are obligate intracellular parasites that usually reside within a non-fusogenic parasitophorous vacuole [1], where they replicate. In the case of *Toxoplasma gondii* (*T. gondii*), this parasitophorous vacuole is highly permeable for molecules up to 1.9 kDa [2]. While the parasite expresses several transporters facilitating uptake of nutrients [3], the role of endocytic uptake mechanisms is unclear.

Coat-dependent intracellular vesicle trafficking is an evolutionary conserved feature of eukaryotic cells to maintain a continuous cargo flow between the plasma membrane and intracellular compartments which ensures their integrity and exchange with their environment [4]. Transport vesicle formation on donor membranes depends on coat proteins which selectively incorporate cargo into vesicles and provide a scaffold for vesicle budding [5]. Clathrin is the prototype vesicle coat protein functioning in protein sorting at endosomal membranes and at the trans-Golgi network (TGN) [6–9]. It forms a three-legged hexameric protein complex in the cytoplasm termed triskelion which represents its functional unit. Triskelia self-assemble at appropriate membranes into a polyhedral clathrate coat which drives clathrin-coated vesicle (CCV) formation and cycle between their assembled membrane-bound and disassembled cytoplasmic state [6]. Clathrin itself is incapable of binding to membranes or cargo directly. Membrane recruitment and cargo specificity rely on compartment-specific adaptor proteins (APs) linking the inner membrane-embedded cargo to the outer clathrin cage [10]. Their specificity to both can be enhanced by additional binding of multiple accessory proteins. AP1 and AP2 are classical adapters interacting with clathrin at the TGN and plasma membrane respectively [11]. Three more AP complexes (AP3-AP5) have been identified to date sharing structural similarities and similarities in complex assembly with AP1 and AP2. Curiously, several highly conserved endocytic factors are absent in apicomplexan parasites, such as the secondary loss of critical components of the ESCRT machinery in several Apicomplexa or multiple losses of adaptins across the phylum [12,13]. Furthermore, recent data demonstrate that factors typically involved in endocytosis and endosomal recycling, such as dynamins [14,15], Rab-GTPases [16-18] and retromer [19] are involved in specialised functions during the biogenesis of the unique apicomplexan organelles, such as the secretory organelles (micronemes and rhoptries) or the Inner Membrane Complex (IMC). Furthermore, caveolin-dependent endocytosis is absent in *T. gondii* [20,21].

**Figure 1:**
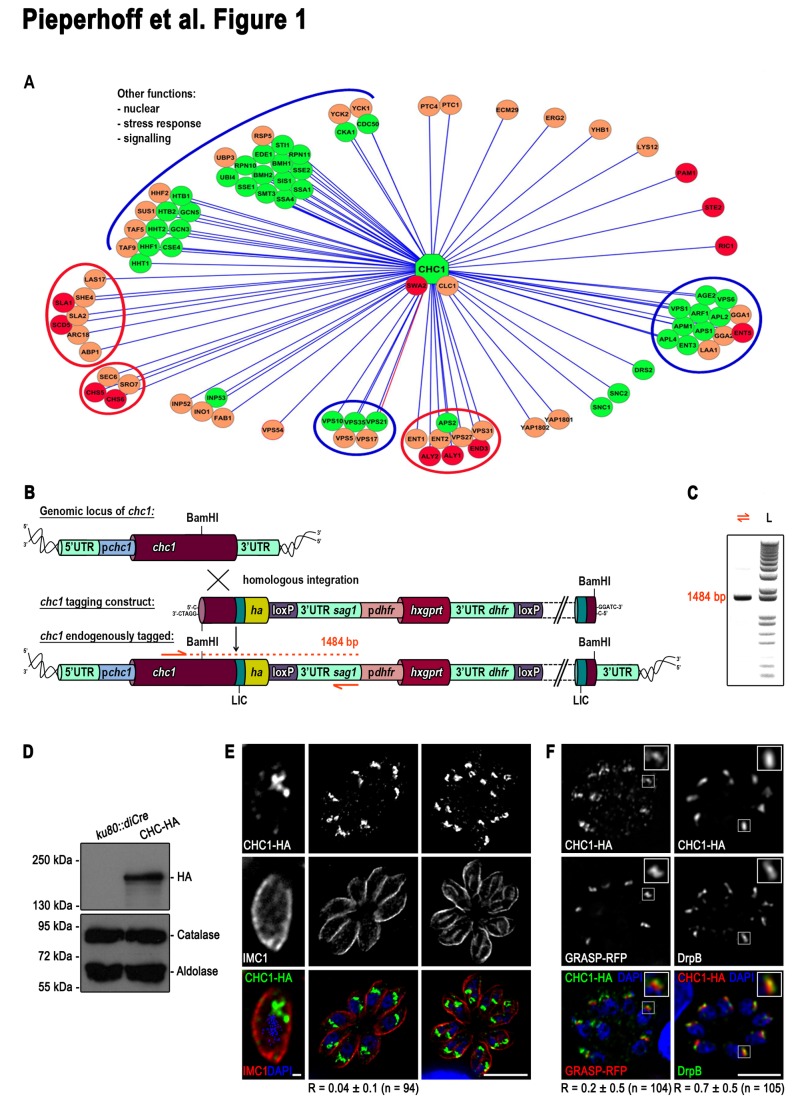
Clathrin interactome and localisation in *T. gondii*. (**A**) Putative clathrin interactome with green symbols conserved in *T. gondii*, orange symbols conserved in most eukaryotes and red symbols indicating fungi specific interactors. For details see also Table S1. (**B**) Schematics of the chc1 endogenous ha tagging construct and its integration into the genomic chc1 locus. UTR, untranslated region. (**C**) Analytical PCR on genomic DNA using oligos indicated as orange arrows in (**B**). The obtained 1484 bp confirms endogenous tagging of *chc1*. (**D**) Immunoblot of indicated parasite lines probed with anti-HA, anti-Catalase, and anti-Aldolase antibodies. Catalase and aldolase were used as loading controls. (**E** and **F**) Immunofluorescence analysis of endogenously *chc1* tagged parasites with indicated antibodies. CHC1-HA shows no colocalisation with IMC1 (**E**) and predominantly accumulates apical to the nucleus (**E** and **F**). It colocalises with DrpB and resides like DrpB adjacent to the trans-Golgi weakly overlapping with GRASP-RFP. (**F**). R, Pearson correlation coefficient ± SD averaged for the indicated number (n) of parasites. Scale bars represent 1 μm and 10 μm in (**E**) and 10 μm in (**F**). Inlets show twofold enlargements of the indicated area.

Therefore, the existence of endocytosis in apicomplexan parasites is still a matter of debate. In *T. gondii*, several reports claim the need of endocytic mechanisms to take up metabolites and fluid-phase markers [22-25] possibly via the micropore [26], a cytostome-like structure. However, no mechanistic data have been described so far and there is scant evidence of clathrin being involved. In *T. gondii* clathrin-like coats have been repeatedly observed by electron microscopy on vesicles at the TGN [27] ( D.J.P. Ferguson, personal communication; see also Figure 1 Fi). In malaria parasites Golgi derived CCVs have been described by ultrastructural analysis [28]. 

To shed light on this controversy and to build a bridge to current findings in other eukaryotes, we studied clathrin localisation and function in *T. gondii*. We generated mutants conditionally expressing a dominant negative version of CHC1, the so called Hub fragment [6]. We demonstrate that clathrin is predominantly found at the TGN with no obvious localisation at the surface including the micropore. Upon expression of Hub we find that protein trafficking through the Golgi/TGN is blocked, resulting in parasites lacking micronemes and rhoptries and with abnormal pellicle formation. Furthermore, mutant parasites exhibit an efficient block in replication, indicating a critical role of CHC1 during mitosis and cytokinesis. In contrast, based on localisation and morphology, we did not find evidence that CHC1 functions in endocytosis.

## Results

### Generation of a putative clathrin interactome suggests a role of CHC1 at the Golgi

We identified a single gene for CHC1 in the genome of apicomplexan parasites that is highly conserved (Figure S1). For example, *T. gondii* CHC1 (TGME49_090950) encodes a predicted protein of 1731 residues (194 kDa) and shows 44% identity to human clathrin. The function and interaction partners of CHC1 have been best studied in ophistokonts, such as yeast, and complex CHC1 interactomes have been established that can be accessed via the Saccharomyces Genome Database (SGD; www.yeastgenome.org). Given that CHC1 functions as a central molecule in vesicle formation of endo- and exocytosis which is conserved in all eukaryotes, we were interested how a putative CHC1 interactome in *T. gondii* relates to the identified interaction factors in yeast (Figure 1 A). All interactors of CHC1 catalogued at SGD as having been confirmed either genetically or physically were used to build a putative interactome. Each interactor was subsequently subjected to multiple BLAST analyses to identify corresponding homologs in *T. gondii* and other apicomplexans. A summary of this comparison is depicted in Figure 1 A, with green symbols conserved in *T. gondii*, orange symbols conserved in most eukaryotes, and red symbols indicating fungi specific interactors. Although it cannot be excluded that some interactors are highly diverged, this analysis suggests that most interactors required for CHC1 function in TGN transport, such as the retromer, are conserved in ophistokonts and apicomplexans. Similarly, interactors required for mitosis and replication are highly conserved (see also Table S2). In contrast, there appears to be a paucity of interactors normally involved in endocytic uptake, such as essential components of the ESCRT complex or actin binding proteins that are required for endocytosis (Figure 1 A). 

### CHC1 localises to the TGN

For the characterisation of *T. gondii* CHC1 we generated a construct that leads to C-terminal tagging with an HA-FLAG epitope tag. Transfection into *Δku80* knockout parasites [29] results in C-terminal tagging of endogenous *chc1*. We confirmed homologous integration by PCR analysis (Figure 1 C). As expected, HA-FLAG tagged CHC1 (CHC1-HA) is expressed with a molecular weight of approximately 195 kDa (Figure 1 D). We performed localisation studies, taking advantage of the HA-FLAG tag and found that CHC1-HA is localised to a region apical to the nucleus indicative of the TGN (Figure 1 E and 1 F). Colocalisation analysis with the Golgi marker GRASP-RFP [30] and DrpB [15] confirmed that CHC1 significantly colocalises with endogenous DrpB (R = 0.7 ± 0.5) and resides like DrpB in juxtaposition but limited colocalisation with GRASP-RFP (R = 0.2 ± 0.5) (Figure 1F). In contrast, we were unable to detect CHC1-HA associated with the surface of freshly invaded or further intracellular developed parasites (Figure 1 D and 1 E). Specifically, no colocalisation with IMC1 has been observed (R = 0.04 ± 0.1) (Figure 1 E). Taken together, these results indicate a role of CHC1 in trans-Golgi network vesicle transport.

**Figure 2:**
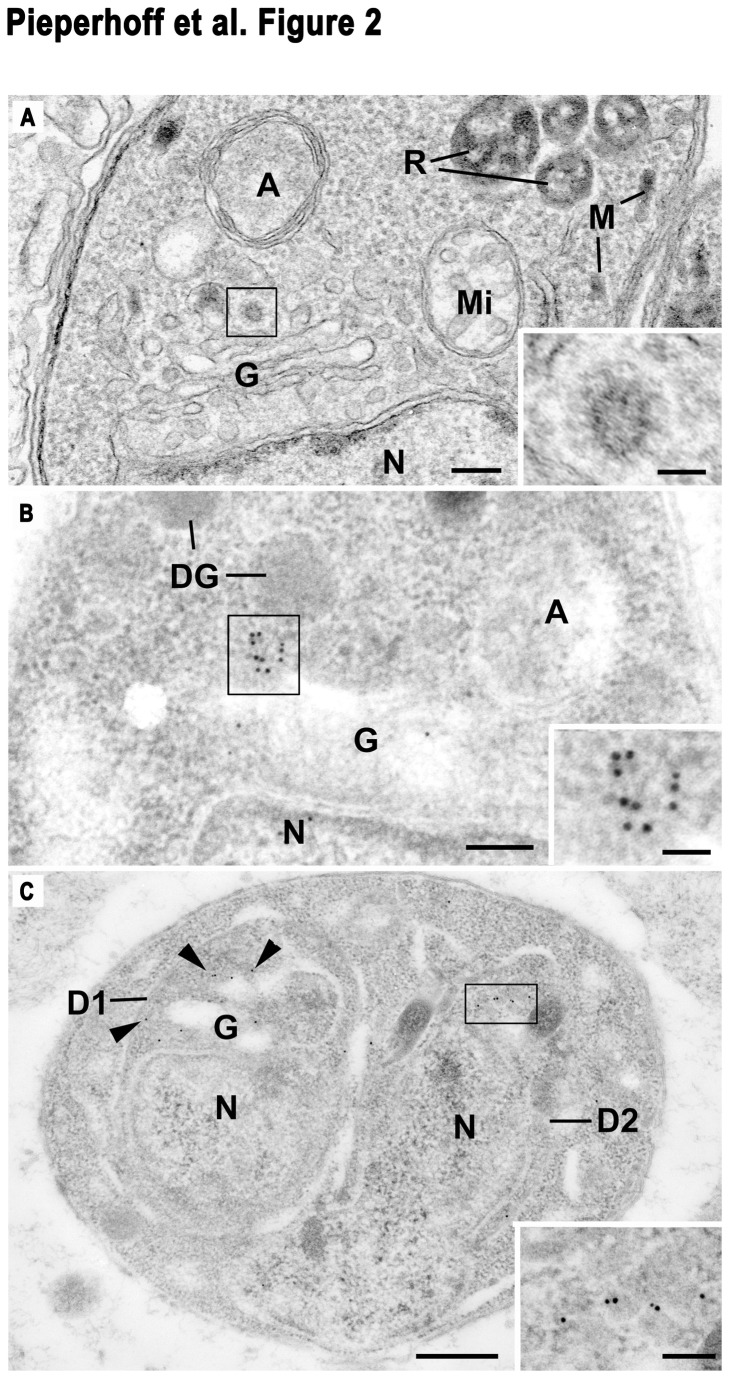
Ultrastructural analysis of CHC-HA expressing *T. gondii* parasites. Routine (**A**) and immuno (**B** and **C**) electron micrographs. (**A**) Section through the anterior of a tachyzoite showing nucleus (N), apicoplast (A), Golgi (G), rhoptries (R), micronemes (M), and mitochondrion (Mi). Scale bar is 100 nm. Insert: Enlargement of the enclosed area showing a coated vesicle. Bar is 50 nm. (**B**) Similar area to that in (**A**) of a parasite processed for immuno electron microscopy showing gold particles located predominately at the periphery of the Golgi (G). N, nucleus; A, apicoplast; DG, dense granules. Bar is 100 nm. Insert: Detail of the enclosed area showing a number of gold particles around a possible vesicle. Bar is 50 nm. (**C**) Section through a parasite undergoing endodyogeny showing the two partially formed daughters (D1, D2) with gold particles (arrowheads) associated with the periphery of the daughter Golgi bodies (G). N, nucleus. Bar is 500 nm. Insert: Enlargement of the enclosed area showing gold particles at the periphery of the Golgi body. Bar is 100 nm.

We analysed the location of clathrin and CCVs in detail by electron microscopy (EM; Figure 2 A). In routinely processed parasites, CCVs were readily identified in the trans-Golgi area but not in other regions of tachyzoites (Figure 2 A). When similar stages were examined by immunoelectron microscopy (IEM) a number of gold particles were localised in the trans-Golgi region (Figure 2 B) coinciding with CCV structures. This was consistent in over 30 appropriately sectioned parasites. A similar location was also observed in daughters during endodyogeny (Figure 2 C). On rare occasions a few gold particles could be identified close to the pellicle (Figure 2 C) but there was no evidence of gold particles associated neither with plasma membranes nor micropores. These observations of clathrin-coated pits and positively stained vesicles at the TGN is in good agreement with light microscopy localisation of CHC1-HA.

### Functional ablation of CHC1 results in multiple defects in organellar biogenesis

**Figure 3:**
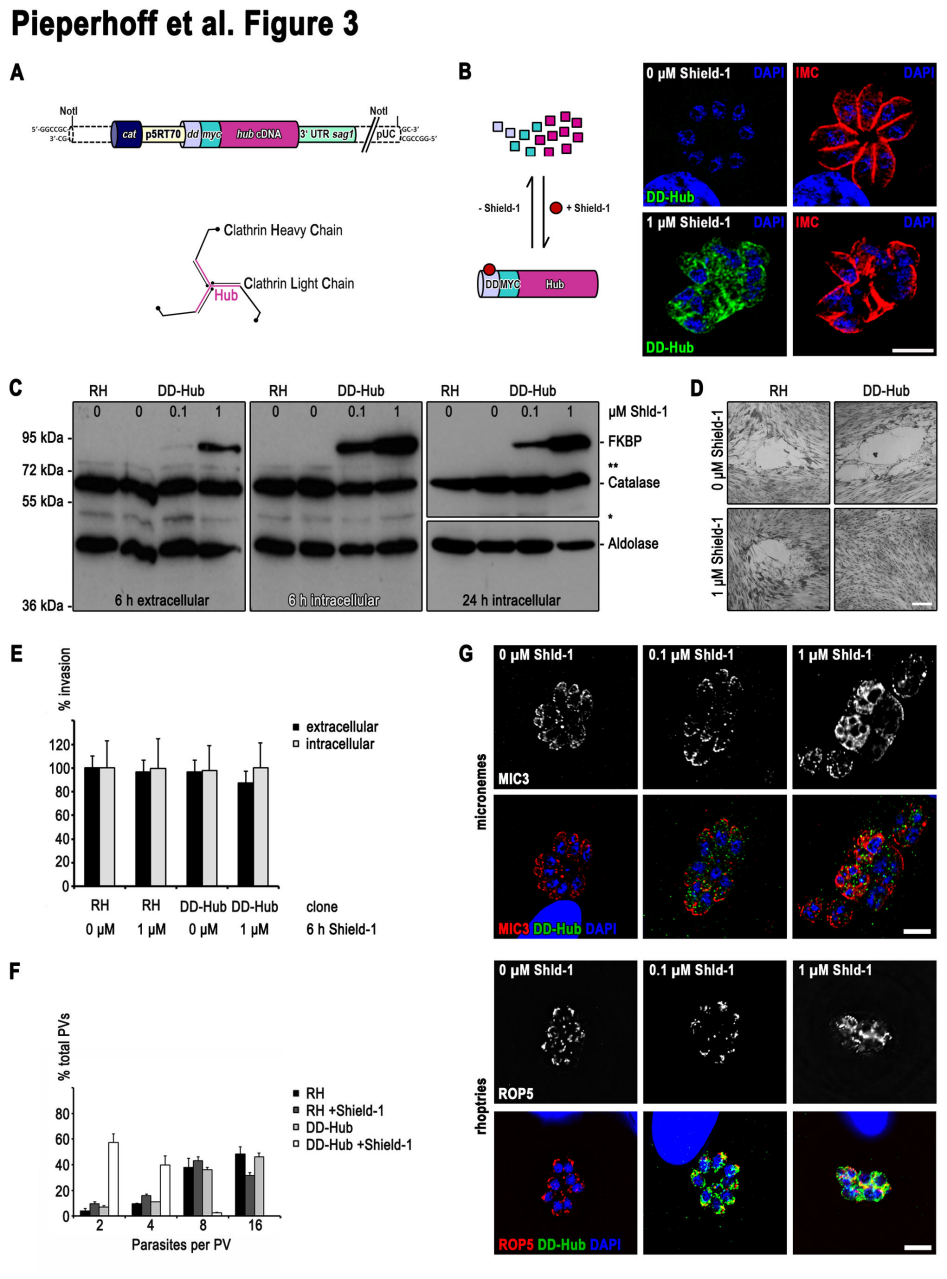
Rapid ablation of CHC1 function in *T. gondii* (**A**) Schematic of the dominant negative DD-Hub construct and of the chlathrin triskelion structure. The Hub fragment is highlighted in pink. *dd*, destabilization domain; *myc*, myc epitope tag; p, promoter; UTR, untranslated region. (**B**) Schematic (left) and immunofluorescence analysis (right) of Shield-1 dependent regulation of DD-Hub. Immunofluoresence has been performed with indicated antibodies 24 hours after incubation of parasites in presence or absence of 1 μM Shield-1. Hub fragments localise to the cytoplasm and their expression lead to deformed IMCs (**C**) Immunoblot of parasite lysates probed with indicated antibodies. Catalase and aldolase were used as loading controls. Intra- or extracellular parasites were treated with 0 μM, 0.1 μM and 1 μM Shield-1 for the indicated time. (**D**) In contrast to the controls DD-Hub expressing parasites show no plaque formation after 7 days incubation in presence of 1 μM Shield-1. Scale bar represents 500 μm. (**E**) For analysis of the invasion rate intra- or extracellular parasites were treated for 6 hr with or without 1 μM Shield-1 prior to the experiment. Data represent mean values of three independent experiments ±SD. (**F**) For analysis of the replication rate indicated parasite lines were cultured in absence or presence of 1 μM Shield-1 and fixed 24 hr post invasion. Data represent mean values of three independent experiments ±SD. (**G**) Immunofluorescence analysis with indicated antibodies of DD-Hub parasites treated with indicated Shield-1 concentrations for 24 hours. Scale bar represents 10 μm. Immunoflourescence images are representative of at least three independent experiments and depicted abnormalities have been observed in 100% of 200 random examined vacuoles compaired to controls.

We took advantage of the ddFKBP-system that allows rapid regulation of protein levels [31] and expressed the CHC1 Hub fragment, that can efficiently block clathrin function in a dominant negative fashion [6,32], N-terminally tagged with ddFKBP-myc (DD-Hub; Figure 3 A and 3 B).

Efficient regulation of DD-Hub was achieved as soon as 6 hours after addition of the inducer Shield-1 as seen in immunofluorescence analysis (Figure 3 B) and western blot analysis of intra- and extracellular parasites (Figure 3 C). As expected, parasite growth was completely blocked upon stabilisation of DD-Hub using 1 μM Shield-1, as confirmed in plaque assays (Figure 3 D). When we analysed the effect of DD-Hub expression in extracellular parasites, we found that host cell invasion is not affected (Figure 3 E), demonstrating that CHC1 and therefore clathrin-mediated endocytosis (CME) and likely receptor-mediated endocytosis (RME) is not involved in host cell invasion. In contrast, DD-Hub expression in intracellular parasites caused abnormal parasite development resulting in a replication arrest at 2-8-cell stage (Figure 3 F). The IMC in dividing parasites was incorrectly formed (Figure 3 B and Figure S2) and parasites were unable to form their unique secretory organelles (Figure 3 G and S2 A). When we analysed host cell egress, we found that egress was completely blocked in malformed parasites (Figure S3 A and S3 B). However, shorter stabilisation times of DD-Hub (6 hours) did not result in a block of induced parasite egress (Figure S3 B).

Together this analysis demonstrates that CHC1 has a predominant function during parasite development inside the host cell, whereas its functional ablation during host cell invasion or egress is tolerated by the parasite.

### CHC1 is essential for Golgi function and segregation

**Figure 4:**
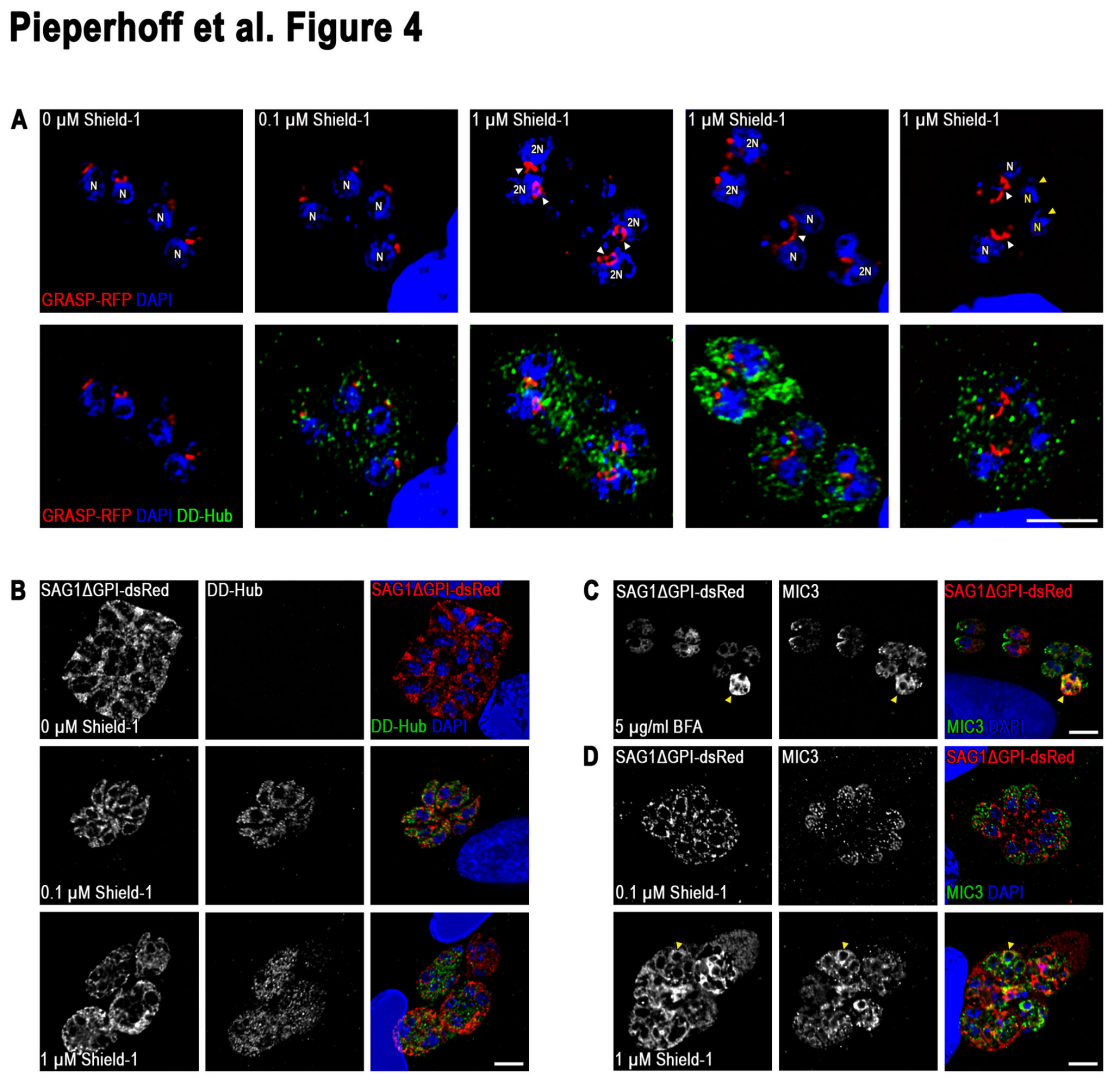
CHC1 is essential for Golgi function and segregation. (**A**) Immunofluorescence analysis of DD-Hub expressing parasites stable transfected with the cis-medial Golgi marker GRASP-RFP cultured for 24 hr in absence or presence of 0.1 and 1 μM Shield-1. Only under high Shield-1 concentrations daughter parasites show abnormal Golgi morphology (white arrow heads) or no Golgi at all (yellow arrow heads, N highlighted in yellow). N, nucleus. (**B**-**D**) Immunofluorescence analysis of DD-Hub expressing parasites stable transfected with SAG1∆GPI-dsRed. After treatment for 24 hr with 0.1 and 1 μM Shield-1 DD-Hub expression causes a block in constitutive secretion of SAG1∆GPI-dsRed. (**C** and **D**) Overnight treatment with 5 μg/ml Brefeldin A (BFA) or 24 hr treatment with 1 μM Shield-1 respectively show an identical block in transport for SAG1∆GPI-dsRed and the micronemal protein MIC3 (arrow heads). Immunoflourescence images are representative of at least three independent experiments and depicted abnormalities have been observed in 100% of 200 random examined vacuoles compaired to controls.

In *T. gondii* the Golgi consists of a single stack apical to the nucleus that is segregated equally during parasite division [33] and this early event is tightly coupled to nuclear division [17,34]. To analyse the behaviour of the Golgi in DD-Hub expressing parasites, parasites were transfected with the Golgi marker GRASP-RFP [30]. While the morphology and segregation of the Golgi is not affected when parasites are incubated in presence of 0.1 μM Shield-1, which leads to only weak stabilisation of DD-Hub (Figure 3 C and 4 A), incubation of parasites in 1 μM Shield-1 led to a significant disturbance of the Golgi. While nuclear division appeared to continue during this incubation time, daughter cells either lacked or inherited an expanded Golgi (Figure 4 A). Next we analysed constitutive secretion using SAG1ΔGPI-dsRed [35] as a marker and confirmed that inoculation of DD-Hub parasites in presence of the inducer Shield-1 leads to a block of constitutive secretion, since SAG1ΔGPI-dsRed accumulates in the early secretory pathway (Figure 4 B). Even weak stabilisation of DD-Hub in presence of 0.1 μM Shield-1 was sufficient to efficiently block constitutive secretion of SAG1ΔGPI-dsRed (Figure 4 B). We observed the same behaviour for the marker proteins MIC3 and SAG1ΔGPI-dsRed, when parasites were treated with Brefeldin A, a drug that specifically inhibits ER to Golgi traffic and leads to disruption of the Golgi (Figure 4 C and 4 D). Under these conditions MIC3 and SAG1ΔGPI-dsRed accumulate in the early secretory pathway and show a significant colocalisation (Figure 4 C and 4 D).

In summary, our colocalisation studies demonstrate that CHC1 is essential for Golgi function, constitutive secretion, and vesicular transport to the IMC, plasma membrane, micronemes and rhoptries. Furthermore, like in other eukaryotes CHC1 appears to be involved in segregation of the Golgi during mitosis [36].

### CHC1 is essential for segregation of endosymbiotic organelles

**Figure 5:**
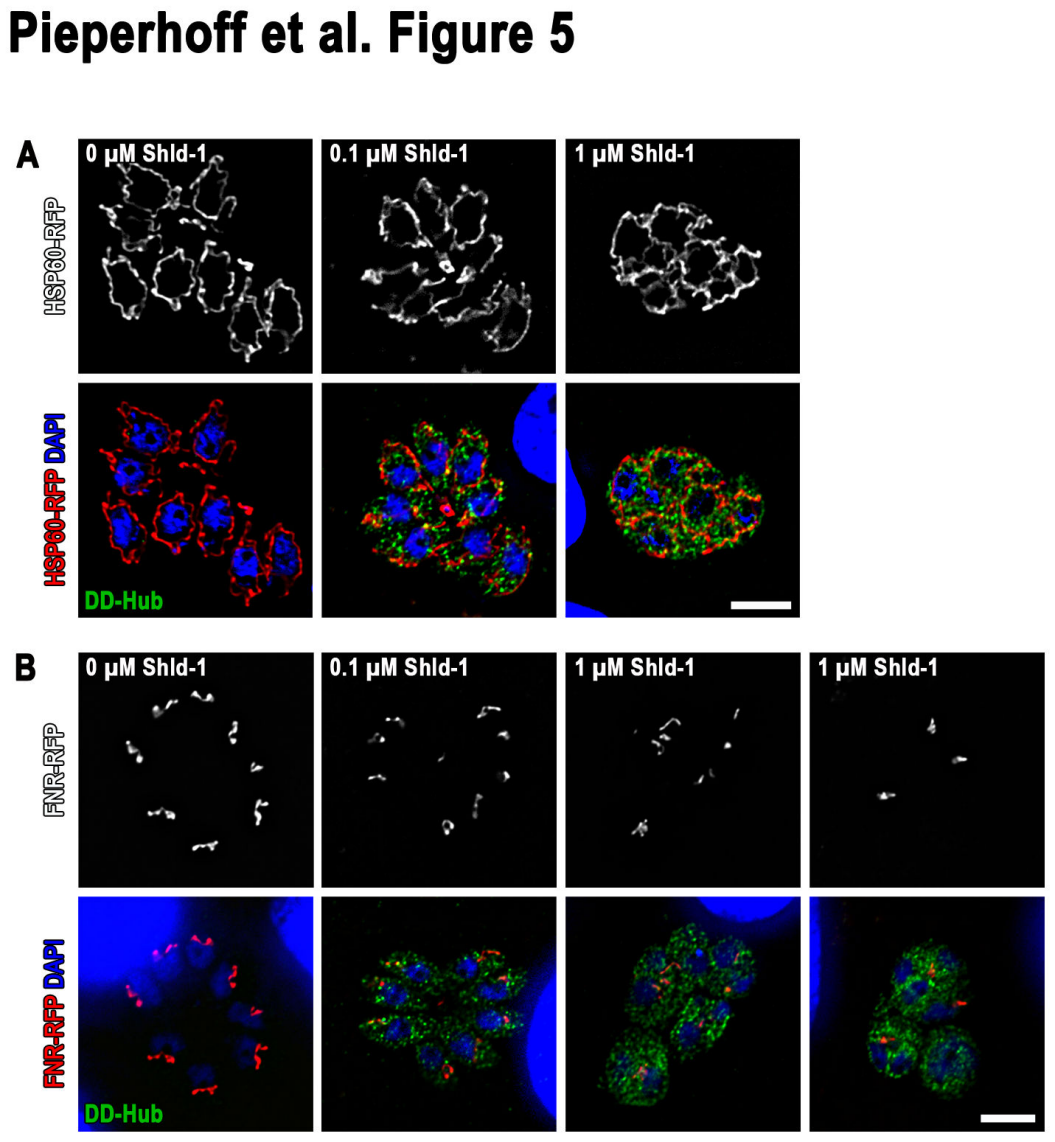
Impact of DD-Hub expression on mitochondrial and apicoplast segregation. Immunofluorescence analysis of DD-Hub expressing parasites stable transfected with either HSP60-RFP (**A**) or FNR-RFP (**B**). Parasites were cultured for 24 hr in absence or presence of 0.1 and 1 μM Shield-1. In presence of high Shield-1 concentrations mutants show mitochondrial and apicoplast segregation defects. Scale bars represent 10 μm. Immunoflourescence images are representative of at least three independent experiments and depicted abnormalities have been observed in 100% of 200 random examined vacuoles compaired to controls.

Since Golgi segregation and replication of the apicoplast is linked in a timely and possibly mechanistic manner [34], we wondered if a block in CHC1 function has an effect on replication and/or segregation of these endosymbiotic organelles. In the case of mitochondrial division, ablation of functional CHC1 affects segregation of mitochondria after mitosis. Each daughter cell appears to contain a mitochondrion, but they are not separated and form a meshwork of connected, aberrant looking mitochondria (Figure 5 A). Similarly, we found that expression of DD-Hub has a significant effect on the segregation of the apicoplast (Figure 5 B). Of note, the observed phenotype for the apicoplast does not correspond to the phenotype obtained by expression of dominant negative dynamin-related protein A (DrpA) [14], indicating that CHC1 and DrpA are not functioning at the same step of apicoplast segregation. Furthermore, we cannot exclude that the block in apicoplast division is merely a downstream effect due to a block of Golgi division or the secretory pathway.

### Ultrastructural analysis of parasites expressing DD-Hub

**Figure 6:**
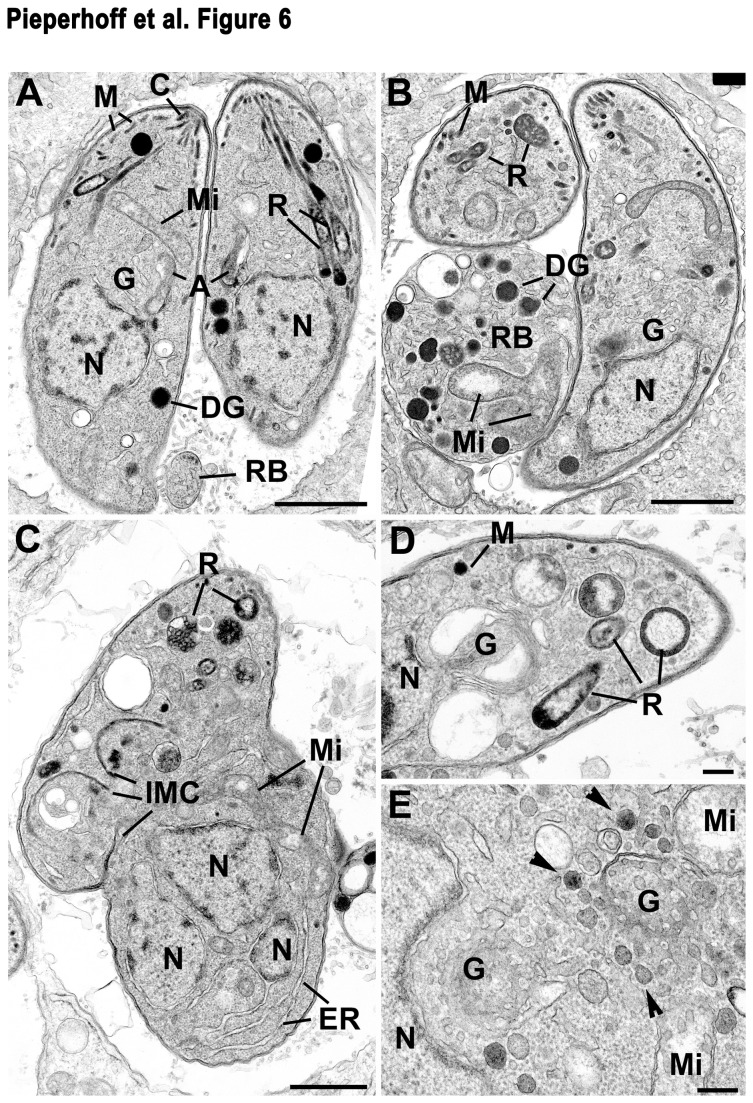
Ultrastructural analysis of stably dominant negative DD-Hub expressing parasites. Parasites were cultured in absence (**A**) or presence (**B**-**E**) of Shield-1 for 24 hrs. (**A**) Section through a parasitophorous vacuole containing two daughters each showing normal morphology with nucleus (N), Golgi body (G) and apical organelles. C, conoid; R, rhoptries; M, micronemes; DG, dense granules. Note the small residual body (RB) is free of organelles. Bar is 1 µm. (**B**) Section of a Shield-1 treated sample showing two daughters enclosed by the double layer pellicle with limited anterior organelles, such as micronemes (M) and rhoptries (R). Note the large residual body (RB), limited by the plasmalemma, containing remnants of parasite organelles consisting of mitochondrion (Mi) and dense granules (DG). N, nucleus; G, Golgi body. Bar is 1 µm. (**C**) Irregularly shaped parasite showing abnormally located daughter inner membrane complexes (IMC), multiple nuclei (N), abnormal pre-rhoptries (R) and convoluted mitochondrion (Mi). Bar is 1 µm. (**D**) Apical end of a daughter exhibiting the nucleus (N), dilated Golgi body (G), rare micronemes (M) and abnormal rhopties (R) which lack ducts. Bar is 100 nm. (**E**) Detail of the perinuclear region showing an expanded Golgi body with a few vesicles with electron dense contents around the periphery (arrowheads). Mi, mitochondrion. Bar is 100 nm.

To verify these results, we performed a detailed ultrastructural analysis of wild type and DD-Hub expressing parasites. In wild type parasites the daughters formed by endodyogeny show normal organellar content and residual body organisation (Figure 6 A). In contrast, the treated parasites showed multiple abnormal features (Figure 6 B - 6 E). At an early stage in treatment, the daughters were relatively normal in shape although certain parasites showed incomplete plasmalemma formation (not shown). In addition, there also appeared to be large residual bodies, identified by being limited by the plasmalemma, containing a number of organelles (Figure 6 B). In later stages the parasites lost their polarised shape and appeared more spherical (Figure 6 C). The cytoplasm of these parasites was more disorganised showing multiple nuclei but without associated daughter formation (Figure 6 C). There were examples of daughter IMC formation but these were atypically located and appeared not to develop (Figure 6 C). There was evidence of early rhoptry formation but these appeared to be disorganised and were unable to undergo maturation with no duct formation (Figure 6 C and 6 D). There were also pleomorphic changes in the appearance of the Golgi region with some appearing dilated (Figure 6 D). In others, the region appeared to be expanded (Figure 6 E). Around certain of the Golgi bodies were a number of vesicles with electron dense contents but no coated vesicles as seen in the wild type (Figure 6 E). The content of these vesicles is similar to that seen in micronemes but cigar-shaped micronemes were rarely observed. In addition, the mitochondrion appeared distributed throughout the cytoplasm and the residual body (Figure 6 B). While the apicoplast could be readily identified in wild type parasites (Figure 6 A), it was rarely seen in treated parasites (Figure 6 B 6 E). The ultrastructural observation of abnormal and disorganised development of various organelles is consistent to that seen by light microscopy. 

## Discussion

In this study we aimed to shed light on the role of endocytosis during the asexual life cycle of *T. gondii*. While it becomes evident that this parasite is capable of using several trafficking factors usually involved in endocytosis or endosomal recycling for the biogenesis of its unique organelles [14,15,17-19,37], the role of endocytosis and RME is controversial. In the absence of caveolin-dependent endocytosis [20,21], clathrin-dependent endocytosis should be the predominant mechanism for endocytic uptake in these parasites.

The importance of clathrin in RME remains unquestioned since its discovery in 1964 [38]. Over the last 50 years a growing number of functional studies have reported evidence for its impact on diverse physiologically processes besides its role in endocytic and secretory membrane transport, such as cytokinesis [8,39,40], Golgi integrity [36], secretory granule biogenesis [41], and mitotic and meiotic spindle organisation and stabilisation [42,43]. Without exception clathrin localisation was always correlated with its site of action in these reports. Hitherto many proteins are presumed to interact with clathrin and/or clathrin-associated proteins purely based on colocalisation although there is no mechanistic evidence excluding a function in alternative endocytic pathways [6]. Clathrin is a long-lived protein with a half-life of 24-36 hours [44]. The development of molecular tools for investigation of clathrin-dependent processes progressed and contributed significantly to the current understanding. Besides traditional molecular techniques, such as RNA interference (RNAi), immunological depletion, and genomic deletion, the dominant negative clathrin inhibitor Hub became accepted as a standard method to study clathrin function. To overcome clathrin turnover the technique has been shown to require 20-40 hrs [45]. Although functional ablation is achieved faster than with other techniques this time frame does still not allow a clear discrimination between short-term and long-term depletion effects since compensatory pathways might become upregulated. 

Clathrin dynamics have been studied predominantly by overexpression of fluorescent fusion proteins although it has been shown that overexpression can cause protein mislocalisation and aggregation, and alter signalling pathways. However, the underlying mechanisms required for its regulation remain still elusive. Endogenous tagging approaches in yeast and mammalian cells strongly support the importance of native protein levels to study such regulatory processes and redefined CME as regular and efficient instead previously described as highly dynamic, inefficient and heterogeneous [46,47]. Clathrin and clathrin-dependent events are thought to be evolutionary conserved even though differences for clathrin requirements have been observed among unicellular and multicellular eukaryotes [48,49]. So far functional studies have been mostly limited to ophistokonts (from yeast to men). Intriguingly, studies performed in the alveolate *Tetrahymena thermophila* suggest that CME might have evolved independently in different phyla [32].

To elucidate clathrin localisation in *T. gondii* we *ha-flag* tagged endogenous *chc1* which allowed visualisation of clathrin by immunoflourescent and immuno goldlabelling. Using this approach we localised CHC1 predominantly at the TGN, confirming previous observations of CCVs at the Golgi [27]. However, we were unable to identify CHC1 at the surface of the parasite or at the limited number of micropores observed. Since clathrin localisation usually correlates with its site of action these results strongly support the assumption of clathrin not being responsible for RME during intracellular development in *T. gondii*.

**Figure 7:**
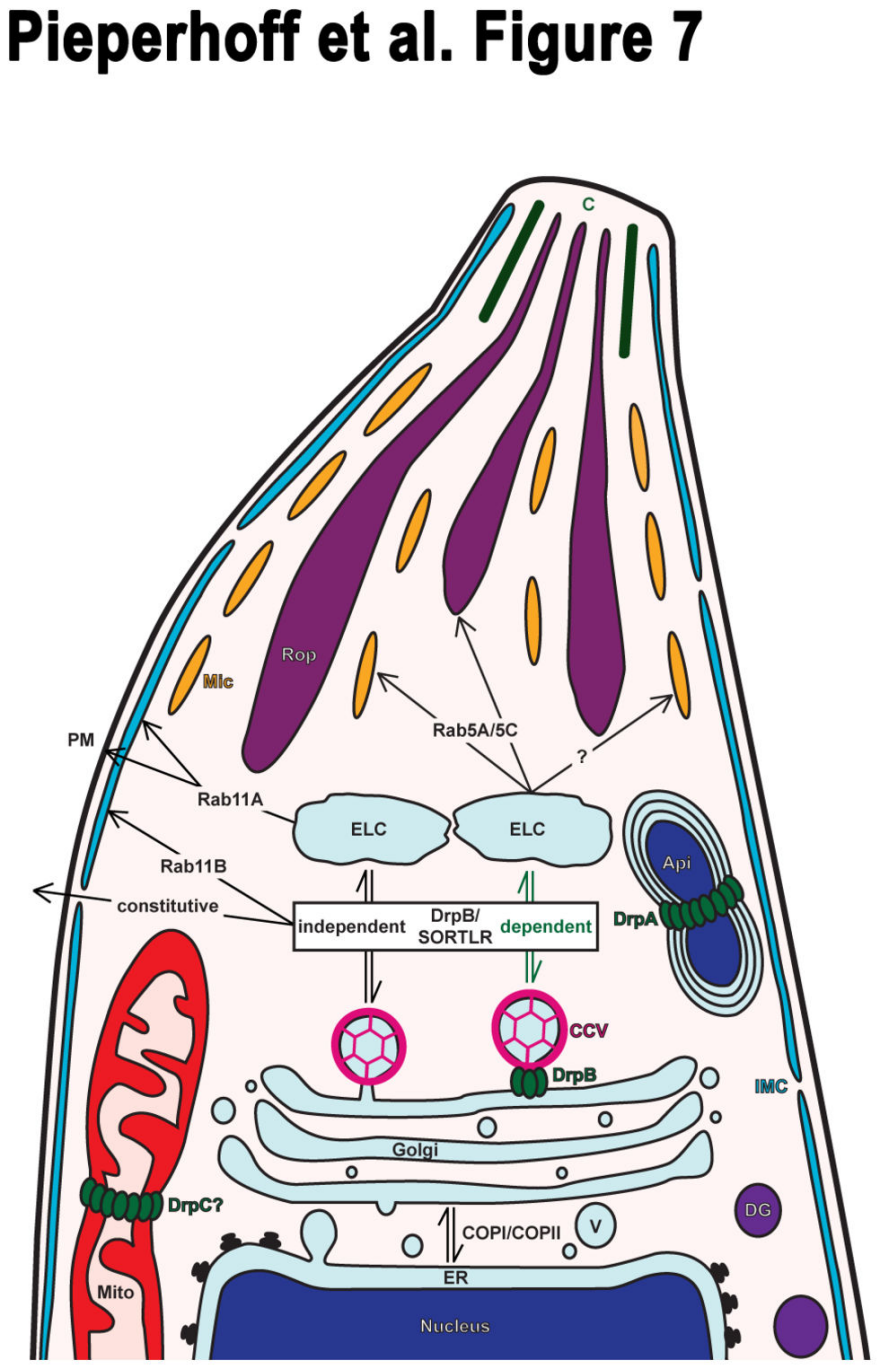
Model of CHC1 function in post-Golgi trafficking and organellar biogenesis in *T. gondii*. Generation of various transport vesicles (V) at the trans-Golgi is reliant on clathrin. In concert with DrpB and SORTLR clathrin is involved in vesicle formation from which secretory organelles such as micronemes (Mic) and rhoptries (Rop) derive. Whereas post-endosome-like compartments (ELC) sorting of secretory vesicles destined for the rhoptries occurs only in a Rab5A/C dependent fashion, targeting to the micronemes occurs cargo specific either dependent or independent of Rab5A/C. In addition, clathrin functions independently of DrpB/SORTLER in constitutive secretion and generation of transport vesicles at the Golgi involved in pellicle biogenesis. Whereby Rab11B mediates inner membrane complex (IMC) formation, Rab11A mediates plasma membrane (PM) formation and is important for IMC maturation. Whereas apicoplast (Api) segregation is regulated by DrpA, fission of mitochondria (Mito) might be regulated by DrpC. A direct function of clathrin in apicoplast and mitochondrial replication remains to be confirmed. CCV, clathrin-coated vesicle; C, conoid; DG, dense granule.

To analyse clathrin function we applied a standard method used in mammalian and plant cells based on overexpression of the CHC1 Hub fragment [45,50,51]. The Hub fragment represents the C-terminal third of the CHC which binds CLCs and can self-assemble with the same kinetics as intact CLCs but polymerises into an open-ended lattice instead of a closed polyhedron [6]. Conditional expression of Hubs in *T. gondii* has been achieved by N-terminal fusion with the destabilisation domain (ddFKBP) [31]. Overall, overexpression of DD-Hub results in a very tight and lethal phenotype, demonstrating an essential role during intracellular replication. We were able to confirm a general, essential function of CHC1 in vesicular budding from the Golgi and found defects in organellar biogenesis (micronemes and rhoptries), formation of the pellicle and aberrations of the apicoplast and mitochondria. Similar phenotypes for the secretory organelles have been observed upon ablation of DrpB, SORTLR, and the small GTPases Rab5A and Rab5C which have been shown to be essential for rhoptry and micronemal biogenesis [15,16,19]. Most likely clathrin acts in concert with DrpB and SORTLR in vesicle formation at the Golgi whereas Rab5A and Rab5C are involved in protein sorting downstream of the Golgi (Figure 7). The resulting replication block and disintegration of the pellicle are reminiscent of the phenotype observed for ablation of Rab11B and Rab11A which play a role in vesicular transport from the Golgi to the IMC and plasma membrane respectively during cytokinesis [17,18]. In addition, clathrin seems to be required for constitutive secretion of SAG1ΔGPI-dsRed which appears to be blocked most probably in the ER upon dominant negative clathrin inhibition. Together, this implies a general role of clathrin at the TGN in vesicle formation which occurs dependent on cargo destination either DrpB-/SORTLR-dependent or -independent (Figure 7) and results in loss of the target organelles. In contrast to mammalian cells *T. gondii* parasites inherit just one Golgi stack that needs to be doubled in size prior to division and equally split into the nascent daughter cells [33]. Interestingly, clathrin depletion resulted in parasites either missing or harbouring an aberrantly appearing, dilated Golgi. We also observed effects on apicoplasts and mitochondria but cannot exclude non-specific, downstream effects at this point. The rapid regulation kinetics of the ddFKBP-system allowed us to over express CHC1 Hub in extracellular parasites to investigate roles of clathrin during gliding motility or host cell invasion. We were unable to find any significant differences, suggesting CME is not involved in these processes.

In summary, our study demonstrates an essential role of CHC1 in Golgi organisation and vesicular transport from the Golgi, while no morphological evidence was found that implicates a function of CHC1 in endocytosis in this parasite. Together with previous studies this reinforces the hypothesis that their endocytic system has been reduced to ensure biogenesis and regulation of their unique organelles. Nevertheless, we cannot exclude that lineage-specific factors might have taken over endocytic roles. It will now be interesting to analyse the role of clathrin interacting factors, such as adaptins, in detail to further our understanding of this unconventional sorting system.

## Materials and Methods

### Generation of putative clathrin interactome

*Saccharomyces cerevisiae* was used as the source organism and physical and genetic interactors were downloaded from SGD database (http://www.yeastgenome.org). All the redundant interactions were removed and the remaining interactions represented as networks using Cytoscape (http://www.cytoscape.org/). The orthologous protein information was obtained by performing pairwise alignment of the sequences using BLASTp at NCBI (http://blast.ncbi.nlm.nih.gov/Blast.cgi). Orthologue groups were obtained from OrthoMCL DB (http://www.orthomcl.org/cgi-bin/OrthoMclWeb.cgi). BLASTp (E-value≤0.05) was performed by taking *S. cerevisiae* protein sequences as queries against *T. gondii* and *P. falciparum* protein sequences obtained from EupathDB (http://eupathdb.org/) and confirming the hit by reverse BLAST. 

### *T. gondii* parasite lines, maintenance and transfections

*T. gondii* RH*∆hxgprt* and *Δku80* [29] tachyzoites were maintained by serial passage in human foreskin fibroblast (HFF) monolayers cultured in Dulbecco’s modified Eagle’s medium (DMEM) supplemented with 10% fetal bovine serum (FBS), 2 mM glutamine, and 25 μM gentamycine at 37°C and 5% CO_2_ in a humidified incubator. Transfections were carried out by electroporation as previously described [52] using approximately 10^7^ freshly egressed or mechanically released parasites of the respective parasite strain.

### Generation of *T. gondii* expression constructs and stable *T. gondii* parasite lines

For C-terminal HA-FLAG epitope endogenous tagging of the *chc1* gene the 3′ flank of the *chc1* gene upstream of the STOP condon was amplified by polymerase chain reaction (PCR) from *T. gondii* RH∆hxgprt strain genomic DNA using oligo pair LIC-CHC1-sense/LIC-CHC1-antisense and inserted into pLIC-HA-FLAG-HXGPRT by ligation-independent cloning (LIC) strategy [53]. 15 μg of the resultant CHC1-HA-FLAG-HXGPRT (CHC1-HA) plasmid were linearised by BamHI within the region of homology for efficient homologous recombination and were transfected into *Δku80* parasites [29]. The resultant transfectants were selected for clonal lines expressing CHC1-HA in presence of 25 μg/ml mycophenolic acid and 40 μg/ml xanthine as previously described (Donald et al. 1996) and subsequently cloned by limiting dilution. Specific integration was confirmed by analytical PCR on genomic DNA using oligo pair CHC1-integration-sense/CHC1-integration-antisense.

 For the generation of the dominant negative CHC1 expression construct p5RT70DDmycHub-CAT (DD-Hub) the coding sequence of the CHC1 Hub fragment was PCR amplified using oligos Hub-sense and Hub-antisense. The resultant PCR fragment was cloned into plasmid p5RT70DDmycYFP-CAT [31] via AvrII and PacI restriction sites whereby YFP was replaced by the Hub coding sequence which was thereby placed under control of the ddFKBP-system. RH*∆hxgprt* parasites were transfected with 60 μg of the resultant plasmid linearised with NotI and co-transfected with 30 μg of NotI linearised pDHFR-TSc3 plasmid [54] conferring resistance to pyrimethamine. Clonal parasite lines expressing DD-Hub were selected in presence of 1 μM pyrimethamine as previously described [54] and subsequently cloned by limiting dilution. For colocalisation experiments clonal parasites were transfected with 30 μg NotI linearised RnGRASP-RFP-CAT (GRASP-RFP) [30], TgHSP60-RFP-CAT (HSP60-RFP) [14], TgFNR-RFP-CAT (FNR-RFP) [55], SAG1∆GPI-dsRed-CAT (SAG1∆GPI-dsRed) [35], or 30 μg HindIII linearised TgGap50-YFP-CAT (GAP50-YFP) [56] respectively and each co-transfected with 15 μg of column purified p5RT70mycGFP-HX digested with KpnI and PacI to remove the p5RT70 promoter and the myc-GFP coding sequence from the HXGPRT cassette comprising plasmid backbone. Clonal parasite lines expressing the respective fluorescent markers were selected in presence of 25 μg/ml mycophenolic acid and 40 μg/ml xanthine as previously described [57] and subsequently cloned by limiting dilution. Oligonucleotide sequences are provided in Table S2.

### Fluorescence and immunofluorescence microscopy of *T. gondii* parasites

For microscopic analysis confluent monolayers of HFF cells seeded onto 13 mm diameter glass cover slips were infected with the respective parasite line and incubated in absence or presence of 0.1 μM Shield-1, 1 μM Shield-1, or 5 μg/ml brefeldin A (BFA) at normal growth conditions.

For immunofluorescence analysis cells were fixed after 24 h either with 4% w/v paraformaldehyde in PBS for 20 min at room temperature or with absolute methanol pre-cooled to -20°C for 20 min at -20°C. Fixed cells were permeabilised with 0.2% Triton X-100 in PBS for 20 min and blocked with 3% w/v bovine serum albumin (BSA) in permeabilisation buffer for 20 min. Cells were incubated for 1 h at room temperature with anti-HA-Tag (6E2) mouse monoclonal antibody (Cell Signaling Technology, Inc.) diluted 1:100, anti-HA-Tag (3F10) rat monoclonal antibody (Roche) diluted 1:500, anti-ddFKBP12 rabbit polyclonal antibody (Thermo Scientific) diluted 1:500, anti-TgIMC1 rabbit antiserum [58] diluted 1:1000, anti-TgSAG1 (DG52) mouse monoclonal antibody diluted 1:1000, anti-TgMIC2 (6D10) mouse monoclonal antibody [59] diluted 1:100, anti-TgMIC3 (T42F3) mouse monoclonal antibody [60] diluted 1:100, anti-TgNtRop5 (T53E2) mouse monoclonal antibody [61] diluted 1:1000, anti-Rop2/3/4 (T34A7) mouse monoclonal antibody [62] diluted 1:1000, or anti-DrpB rat monoclonal antibody (a kind gift from Peter Bradley) diluted 1:500 in blocking buffer. Cells were washed three times with PBS and incubated for 1 h at room temperature with Alexa Fluor 488-, Alexa-594- and/or Alexa Fluor 350-conjugated secondary antibodies (Molecular Probes, Invitrogene) diluted 1:3000 in blocking buffer. Cover slips were washed with PBS, rinsed with water and mounted onto microscope slides with Dapi-Fluoromount-G (SouthernBiotech). Z-stack images of 0.2 µm increments were collected on a DeltaVision Core epifluorescence microscope (Applied Precision, GE) fitted with a 100x oil immersion lens (UPlanSApo, NA 1.40) lens and equipped with a Photometrics CoolSNAP HQ2 CCD camera using Applied Precision softWoRx Suite 2.0 software. Deconvolution was performed with Applied Precision softWoRx Suite 2.0 software and deconvolved images were further processed with ImageJ 1.44 and Adobe Photoshop CS4 Extended software. Images are representative of at least three independent experiments. Per condition 200 vacuoles were examined with a Carl Zeiss Axiovert 40 CFL inverted epifluorescence microscope fitted with a 100x oil immersion lens (A-PLAN, NA 1.25) and mean values of 3 independent experiments ± SD were determined unless stated otherwise To measure the degree of colocalisation the Pearson correlation coefficient (R) of deconvoluted z stacks was calculated using Imaris v7.6.1 image analysis software. 

### Immunoblot analysis of total *T. gondii* parasite lysates

For immunoblot analysis parasites were cultivated in absence or presence of 50 nm rapamycin for 36 or 48 h. Freshly egressed or mechanically released parasites were harvested, washed once in ice cold PBS, and resuspended in Laemmli sample buffer containing 2% SDS and 100 mM DTT. Total parasite lysates were boiled for 5 min at 95°C and spun down for 5 min at 21.100 x g at room temperature. Proteins of total lysates of either 2.5 or 5 x 10^6^ parasites were separated on 6 or 12% sodium dodecyl sulphate polyacrylamide gels at 200 V, and transferred onto nitrocellulose membranes. Membranes were blocked with 3% w/v dried skimmed milk and 0.2% Tween 20 in PBS and probed with anti-HA-Tag (6E2) mouse monoclonal antibody (Cell Signaling Technology, Inc.) diluted 1:500, anti-c-Myc (9E10) mouse monoclonal antibody (Santa Cruz Biotechnology, Inc.) diluted 1:500, anti-ddFKBP12 rabbit polyclonal antibody (Thermo Scientific) diluted 1:500, anti-TgCatalase (#84) rabbit antiserum [63] diluted 1:3000, and anti-TgAldolase (WU1614) polyclonal antibody [64] diluted 1:10.000 in blocking buffer. Primary antibodies were detected with Peroxidase-conjugated AffiniPure Goat Anti-Rabbit IgG (H+L) and/or Peroxidase-conjugated AffiniPure Donkey Anti-Mouse IgG (H+L) secondary antibodies (Jackson ImmunoResearch Laboratories, Inc.) respectively diluted 1:50.000 in blocking buffer containing 0.4% Tween 20. For detection immunoblots were treated with Amersham ECL Plus Western Blotting Detection Reagents and exposed to Kodak General Purpose Blue Medical X-Ray Films. Images are representative of at least three independent experiments.

### Electron microscopy

Monolayers of HFF, grown on 6 cm dishes, were infected with DD-Hub parasites and cultured for 12, 24 and 36 h in absence or presence of 1 mM Shield-1 and subsequently fixed with 2.5 % gluteraldehyde in 0.1 M phosphate buffer pH 7.4 (1M Na_2_HPO_4_, 1M NaH_2_PO_4_). Samples were processed for routine electron microscopy as described previously [65]. In summary, samples were post-fixed in osmium tetroxide, dehydrated, treated with propylene oxide and embedded in Spurr’s epoxy resin. Thin sections were stained with uranyl acetate and lead citrate prior to examination in a JEOL 1200EX electron microscope.

CHC-HA parasite samples for immunoelectron microscopy were fixed with 2% paraformaldehyde in 0.1 M phosphate buffer pH 7.4 and processed as described previously [66]. In summary, the samples were dehydrated and embedded in LR White resin. Then sections were floated on drops of 1% BSA in PBS to block non-specific staining and then incubated with anti-HA-Tag (6E2) mouse monoclonal antibody (Cell Signaling Technology, Inc.), washed and exposed to goat anti-mouse IgG conjugated to 10 nm gold particles. Sections were stained with uranyl acetate prior to examination in the electron microscope.

### Plaque assay

For growth and viability analysis of the respective parasite lines HFF monolayers, grown in six-well cell culture plates, were infected with 50 parasites per well and cultured for 7 days in absence or presence of 1 μM Shield-1 under normal growth conditions. Cells were fixed with absolute methanol pre-cooled to -20°C for 10 min at room temperature, stained for 30 min with Giemsa and the latter intensely washed off with PBS. Images were captured with a Nikon Coolpix 5400 digital camera attached to a Nikon SMZ 1500 binocular fitted with a Nikon MXA 5400 objective. Images are representative of at least three independent experiments.

### Invasion assays

Prior to invasion analysis parasites of the respective parasite line were treated intra- and extracellular for 6 h with and without 1 μM Shield-1 under normal growth conditions. Subsequently, pulse invasion experiments were performed as described earlier [67]. In brief, intracellular parasites were mechanically released from the host cells by scraping off the host cells from the cell culture dishes and passing the lysate three times through a 26-gauge syringe needle. Extracellular parasites were spun down at 1000 g for 10 min at room temperature and resuspended in Endo buffer (44.7 mM K_2_SO_4_, 10 mM MgSO_4_, 106 mM sucrose, 5 mM glucose, 20 mM Tris-H_2_SO_4_, 3.5 mg/ml BSA, pH 8.2). 13 mm diameter glass cover slips covered with confluent HFF monolayers were washed once with Endo buffer and infected with 5 x 10^6^ parasites each. After 20 min incubation at normal growth conditions Endo buffer was gently replaced by invasion medium (DMEM completed with 10 mM HEPES and 3% FBS) pre-warmed to 37°C. After 30 min incubation at normal growth conditions extracellular parasites were washed off with PBS and cells were fixed with 4% w/v formaldehyde and 0.05% v/v glutaraldehyde in PBS for 20 min at room temperature. Parasites were immunolabelled with anti-TgSAG1 (T4 1E5) mouse monoclonal antibody [67] diluted 1:1000 prior to permeabilisation to mark non-invaded parasites, with anti-ddFKBP12 rabbit polyclonal antibody (Thermo Scientific) diluted 1:500 after permeabilisation to check for DD-Hub expression, and nuclei were stained by mounting with Dapi-Fluoromount-G (SouthernBiotech). Percentage of numbers of invasion events were determined in 20 fields of views using a Carl Zeiss Axiovert 40 CFL inverted epifluorescence microscope fitted with a 100x oil immersion lens (A-PLAN, NA 1.25) and mean values of three independent experiments ± SD were plotted on a bar graph with scored numbers for non-treated RH*∆hxgprt* parasites defined as 100% invasion.

### Replication analysis

For replication analysis confluent HFF monolayers grown on 13 mm diameter glass cover slips were infected with 10^6^ freshly egressed or mechanically released parasites of the respective parasite strains. Parasites were spun onto host cells at 500 x g for 5 min at room temperature and were allowed to invade host cells for 1 h at normal growth conditions. Cover slips were dipped four times into PBS to wash off non-invaded parasites, transferred into new 24-well cell culture plates, and incubated for 24 h in absence or presence of 1 μM Shield-1. Subsequently, cells were fixed with 4% w/v formaldehyde and parasites were immunolabelled with anti-TgSAG1 (DG52) mouse monoclonal antibody diluted 1:1000 and anti-ddFKBP12 rabbit polyclonal antibody (Thermo Scientific) diluted 1:500, and nuclei were stained by mounting with Dapi-Fluoromount-G (SouthernBiotech). Percentage of parasite numbers of 100 parasitophorous vacuoles were determined per condition with a Carl Zeiss Axiovert 40 CFL inverted epifluorescence microscope fitted with a 100x oil immersion lens (A-PLAN, NA 1.25). Mean values of two independent experiments ± SD were plotted in a bar graph.

### Natural and induced egress analysis

For natural egress 10^5^ and for induced egress 1.5 x 10^6^ freshly egressed or mechanically released parasites of the respective parasite strains were added onto confluent HFF monolayers grown on 13 mm diameter glass cover slips and were allowed to invade for 2 h at normal growth conditions. Non-invaded parasites were removed by three washes with PBS. To analyze natural egress intracellular parasites were further incubated in absence or presence of 0.01 μM, 0.1 μM and 1 μM Shield-1 and fixed with 4% w/v formaldehyde after 24 h, 36 h, 48 h, 72 h, 96 h and 120 h. To analyse induced egress samples were further incubated for 32 h at normal growth conditions and treated with or without 1 μM Shield-1 for 32 h or 6 h prior to the experiment. Samples were washed once with DMEM pre-warmed to 37°C and DMEM was replaced with DMEM complemented with or without 2 μM A23187 pre-warmed to 37°C in order to artificially induce egress. After incubation for 5 min, 10 min, and 15 min at normal growth conditions samples were washed once with PBS, and fixed with 4% w/v formaldehyde. For visualisation parasites were immunolabelled with anti-TgSAG1 (DG52) mouse monoclonal antibody diluted 1:1000, and nuclei were stained by mounting with Dapi-Fluoromount-G (SouthernBiotech). Samples were examined with a Carl Zeiss Axioskope 2 MOT Plus inverted epifluorescence microscope fitted with a 10x objective lense (Plan-APO-CHROMAT, NA 0.75) or a 40x objective lense (Plan-APO-CHROMAT, NA 0.45) respectively, and equipped with a Hamamatsu Photonics Orca-ER CCD camera. Images were acquired with Improvision OpenLab 5.0 software and processed with Adobe Photoshop CS4 Extended software. Images are representative of at least three independent experiments.

## Supporting Information

Figure S1 **CHC1 is highly conserved in apicomplexan parasites.** ClustalW multiple sequence alignment of the putative *T. gondii* CHC1 protein sequence and its orthologues in the indicated species. Accession numbers are the following: *Toxoplasma gondii* (TGME49_090950), *Plasmodium falciparum* 3D7 (PFL0930w), *Plasmodium berghei* ANKA strain (pber|PBANKA_143470), *Plasmodium yoelii* yoelii 17XNL strain (PY01854), *Plasmodium chabaudi chabaudi* (PCHAS_143670), *Plasmodium knowlesi* *strain* H (PKH_143620), *Plasmodium vivax* SaI-1 (PVX_123620), *Theileria* *annulata* strain Ankara (TA04775), Babesia bovis T2Bo (XP_001609347.1), *Neospora caninum* (NCLIV_042820), *Cryptosporidium muris* RN66 (CMU_025200), *Tetrahymena* *thermophila* SB210 (24.m00346), *Schizosaccharomyces pombe* (NP_594148), *Saccharomyces cerevisiae* S288c (scer_s288c__YGL206C), *Cyanidioschyzon merolae* strain 10D (CMF103C), *Dictyostelium discoideum* AX4 (chcA), *Drosophila melanogaster* (FBpp0111710), *Homo sapiens* (ENSP00000269122). The Hub protein sequence used for expression in *T. gondii* is shaded in pink.
(PDF)

Figure S2 **Immunofluorescence analysis of DD-Hub expressing parasites.** Parasites were cultured for 24 hr in presence of indicated Shield-1 concentrations and analysed with indicated antibodies. (**A**) Colocalisations of IMC1 andSAG1 and GAP50-YFP and IMC1 respectively. (**B**) Colocalisations of DD-Hub and MIC2 and ROP2/3/4 respectively. Scale bars represent 10 μm. Immunoflourescence images are representative of at least three independent experiments and depicted abnormalities have been observed in 100% of 200 random examined vacuoles compaired to controls.
(TIF)

Figure S3 **Natural but not induced egress is affected upon DD-Hub expression.** (**A**) Natural egress. Immunofluorescence analysis of DD-Hub expressing parasites cultured for 48 hr in absence and presence of 1 μM Shield-1 and labelled with anti-SAG1-antibody. Inlets show threefold enlargements of the indicated area. Scale bar represents 100 μm. (**B**) Induced egress. Immunofluorescence analysis of DD-Hub expressing parasites grown for 32 hr and labelled with anti-SAG1-antibody. Treatment with 1 μM Shield-1 was either continuously or 6 hr prior to induction of host cell egress with 2 μM calcium ionophore A23187. Scale bar represents 25 μm. Whereas 6 hr incubation with Shield-1 has no influence on egress, long time exposure to Shield-1 results in an egress phenotype. Indicating that functional CHC1 might not play a role in RME and that the egress phenotype is due to the dramatic morphology changes of the parasites (see Figure 3 and 4, and S3). Immunoflourescence images are representative of at least three independent experiments.
(TIF)

Table S1 **Summary of Interactome analysis.**
(XLSX)

Table S2 **Oligos used for cloning of *T. gondii* expression constructs and confirmation of specific construct integration and site specific recombination.**
(DOCX)
