# Supplementary material for: The Role of Clathrin in Post-Golgi Trafficking in Toxoplasma gondii
Source: PLoS One. 2013 Oct 11;8(10):e77620. doi: 10.1371/journal.pone.0077620 (PMC3795686; doi:10.1371/journal.pone.0077620)
Supplement: Figure S1 — CHC1 is highly conserved in apicomplexan parasites. ClustalW multiple sequence alignment of the putative T. gondii CHC1 protein sequence and its orthologues in the indicated species. Accession numbers are the following: Toxoplasma gondii (TGME49_090950), Plasmodium falciparum 3D7 (PFL0930w), Plasmodium berghei ANKA strain (pber|PBANKA_143470), Plasmodium yoelii yoelii 17XNL strain (PY01854), Plasmodium chabaudi chabaudi (PCHAS_143670), Plasmodium knowlesi strain H (PKH_143620), Plasmodium vivax SaI-1 (PVX_123620), Theileria annulata strain Ankara (TA04775), Babesia bovis T2Bo (XP_001609347.1), Neospora caninum (NCLIV_042820), Cryptosporidium muris RN66 (CMU_025200), Tetrahymena thermophila SB210 (24.m00346), Schizosaccharomyces pombe (NP_594148), Saccharomyces cerevisiae S288c (scer_s288c__YGL206C), Cyanidioschyzon merolae strain 10D (CMF103C), Dictyostelium discoideum AX4 (chcA), Drosophila melanogaster (FBpp0111710), Homo sapiens (ENSP00000269122). The Hub protein sequence used for expression in T. gondii is shaded in pink. (PDF) [file pone.0077620.s001.pdf]

```
                        10        20        30        40        50        60        70        80        90       100       110       120
                ....|....|....|....|....|....|....|....|....|....|....|....|....|....|....|....|....|....|....|....|....|....|....|....|
T. gondii       ----MAQQFPIQLHSVVNLADQGVSASSFRFGNVAMHGDKNLVVKDT------------------ETNELFIFSLSVREVHEGRVSPSSPGFSFTKKPTQAEAALMHPSEKVVALRAKTE
P. falciparum   ----MSQNNPLSVCVADNLINYDIQNESFRLGNVSVEGDKYICVKEN------------------VNENTQVVVINLHNKNS------------TRKHMKAESVIIHPNDPILALKGTIK
P. berghei      ----MSSNSPISVNLVDNLSSYNIQNESFRLGNVSIEGDKFICVKEN------------------VNDNTQVVVINLYNQVS------------IRKYMKADSVIIHPNDPILALRGSIK
P. yoelii       ----MSSNSPISVSLVDNLASYNIQNESFRLGNVSIEGDKFICVKEN------------------VNDNTQVVVINLYNQVS------------IRKYMKADSVIIHPNDPILALRGSIK
P. chabaudi     ----MSSNSPLSVTLVDNLASYNIQNESFRLGNVAIEGDKFICVKEN------------------VNDNTQVVVINLYNQIS------------IRKYMKADSVIIHPNDPILALRGSIK
P. knowlesi     ----MNPNNPLSVAVADSLSAYDIQNESFRLGNVSIEGDKYICVKEN------------------VNENTQVVVINLQNKIS------------TRKYMKAESVIIHPNDPILALRGTIK
P. vivax        ----MNPNNPLSVAVADSLSAYDIQNESFRLGNVSIEGDKFICVKEN------------------VNENTQVVVINLQNKIS------------TRKYMKAESVIIHPNDPILALRGTIK
T. annulata     -----MNNYPIITNTILNLRNLGFVDNNFKFDVLSL-EDRYISIKEQ------------------DGDNLTVAIIDLYNNNS-----------IIRKPMKAEAAIMNPNKPIIALRAKLD
B. bovis        -----MAGAPVKINTLLRLNSLGFKDGCFRFGALTLGGDRFVCIRES------------------DESSHSVSIIDLYNGNE-----------VSRRPIKAESTIMNPHKPIIALKASIQ
N. caninum      ----MAQQFPIQLHSVVNLADQGVSASSFRFGNVAMHGDKNLVVKDT------------------ESNELYIFSLAVREVQEGRVSPSLPAFSFTKKPTQAEAALMHPSEKIVALRAKTE
C. muris        MVIHMKGGVPIAANILVNLEELGIGSSCFRFGSVTLEGDKYVGVKETS-----------------VDGGSQIVIVDTSTKHIN------------RKPMKAESALIHPLENILVVRGQSE
C. parvum       --MQNKGGIPITTNVLANLEELGINSSCFRFGSLTLEGDKYVGVKETS-----------------VDGGSQIVVIDTQSKGIN------------RKPMKAESALIHPIENILVVRGRYE
C. hominis      --MQNKGGIPITTNVLANLEELGINSSCFRFGSLTLEGDKYVGVKETS-----------------VDGGSQIVVIDTQSKGIN------------RKPMKAESALIHPIENILVVRGRYE
T. thermophila  ----MQGGVPIRVQTVASLTQLGVGSEYLKFNQTSFQSDRTICINDKNAHLKEQEPYHKPVELQAQPKNQRIILQIVDNNFS-----------VNRKPNKAEYSLNHPTKNILSMRAPSP
S. pombe        ----MAQQLPIRFSEVLQLASVGIQPSSFGFANVTLESDKYVCVRD-----------------NPN-GVNQVVIVDLEDPSN-----------VLRRPISADSVILHPKKKIIALKAQ-R
S. cerevisiae   -----MSDLPIEFTELVDLMSLGISPQFLDFRSTTFESDHFVTVRE-----------------TKD-GTNSVAIVDLAKGNE-----------VTRKNMGGDSAIMHPSQMVISVRANGT
C. merolae      -----MAAAPVVFEELFTLPSVGISPTHISFSNVTMESDRVICVRE-----------------TSD-QGNKLAIIDVTKPDQ-----------IVRRPITADSAIMNPESRIIALRAGAQ
D. discoideum   -----MTNLPIRFQEVLQLTNLGIGSNSIGFSTLTMESEKYICIRE-----------------TTPDDKNNVVIIDTDNPSQ-----------ILRKQMKTDAAIMNPKEPILALKIGQV
A. thaliana     ---MAAANAPIIMKEVLTLPSVGIGQQFITFTNVTMESDKYICVRE-----------------TAP--QNSVVIIDMNMPMQ-----------PLRRPITADSALMNPNSRILALKAQVP
D. melanogaster ----MTQPLPIRFQEHLQLTNVGINANSFSFSTLTMESDKFICVRE-----------------KVN-DTAQVVIIDMNDATN-----------PTRRPISADSAIMNPASKVIALKAQKT
H. sapiens      ----MAQILPIRFQEHLQLQNLGINPANIGFSTLTMESDKFICIRE-----------------KVG-EQAQVVIIDMNDPSN-----------PIRRPISADSAIMNPASKVIALKAGKT

                       130       140       150       160       170       180       190       200       210       220       230       240
                ....|....|....|....|....|....|....|....|....|....|....|....|....|....|....|....|....|....|....|....|....|....|....|
T. gondii       GSA----------------------------------GHMIQVLNLETKVRLGTAQMNE-PVVYWRWVAPTLLALVTDRAVYHWTVGEN-GETTAEP-----------------------
P. falciparum   NMN----------------------------------TIFLQVFNIETKEKICSLNLNE-YMNYWKWINNDTIAIVCEKNVYHWNIDIHNTKKNKDNNDNNNNNNNNNNNNNNNNYFNTL
P. berghei      NAN----------------------------------TIFLQVFNIETKEKICSLNLNE-HINYWKWINNDTIAIVCEKNVYHWNIDMYNTKKNKEN--------------------NVL
P. yoelii       NAN----------------------------------TIFLQVFNIETKEKICSLNLNE-HINYWKWINNDTIAIVCEKNVYHWNIDMYNTKKNKEN--------------------NVL
P. chabaudi     NAN----------------------------------TIFLQVFNIETKEKICSLNLNE-PINYWKWINNDTIAIVCEKNVYHWNIDMYNTKKNKEN--------------------NVL
P. knowlesi     NVN----------------------------------TIFLQVFNIETKEKICSLNLNE-YMNYWKWINNDTIAIVCEKNVYHWNIDIHNSKKHKEN--------------------YTL
P. vivax        NVN----------------------------------TIFLQVFNIETKEKICSLNLNE-YMNYWKWINNDTIAIVCERNVYHWNIDIHNSKRHKEN--------------------YTL
T. annulata     NNY------------------------------------SIQVYLIYT--NFSYYQFDQ-RIIYWKWLNNMELVIITETLVYHWMIGSN-PKYMFEL-----------------------
B. bovis        NGH------------------------------------FIQVFHLETKEKIGTHQFTE-SVVFWNWISPTKLGIVTDNSVYHWNIGSEEPVLIFNR-----------------------
N. caninum      GSA----------------------------------GHMIQVLNLETKARLGTAQMSE-PVVYWRWVGPNLLALITDRTVFHWTVGENNTETSAEP-----------------------
C. muris        VNG-----------------------------------CTVQIFNLGSKEKLGAFVFPE-SIVYWRWLNSRILAIIGDKAVYHWTINGG-SNTLPLK-----------------------
C. parvum       DNG-----------------------------------CTVQIFNLDSKEKLGAFLFPE-SVVFWRWLTPRILAIVGDKGIYHWTIESGNSNSIPVR-----------------------
C. hominis      DNG-----------------------------------CTVQIFNLDSKEKLGAFLFPE-SVVFWRWLTPRILAIVGDKGIYHWTIESGNSNSIPVR-----------------------
T. thermophila  NGVGCYVQRGEKFQIFSSFLLKQSLFFPCFQTLINGVQQNIKIYNLDNREKLKHIDLNE-NIVYMTWVNNNILAMVTPTKVYHSDLTS---QAPSTV-----------------------
S. pombe        ---------------------------------------QLQVFDLEAKAKINSYVMNQ-DVVYWTWISDSVIGMVTDTSVFHWTVSG----SDPVK-----------------------
S. cerevisiae   ---------------------------------------IVQIFNLETKSKLKSFTLDE-PVIFWRWLSETTLGFVTARSILTSNVFDGNVNAKPQL-----------------------
C. merolae      ----------------------------------------IQLFDITSKTRIKSYLLSEGDIVFWKWLSGRTLGIVTANAVYSWSIDASS-TAPPAK-----------------------
D. discoideum   ----------------------------------------LQLISIEQKMQLKSCQMQE-PLEFWKWISPNTLALVTATSVFHWTKEG---NSDPVK-----------------------
A. thaliana     GTT----------------------------------QDHLQIFNIEAKAKLKSHQMPE-QVAFWKWITPKMLGLVTQTSVYHWSIEG---DSEPVK-----------------------
D. melanogaster ----------------------------------------LQIFNIEMKSKMKAHTMNE-DVVFWKWISLNTLALVTETSVFHWSMEG---DSMPQK-----------------------
H. sapiens      ----------------------------------------LQIFNIEMKSKMKAHTMTD-DVTFWKWISLNTVALVTDNAVYHWSMEG---ESQPVK-----------------------

                       250       260       270       280       290       300       310       320       330       340       350       360
                ....|....|....|....|....|....|....|....|....|....|....|....|....|....|....|....|....|....|....|....|....|....|....|
T. gondii       EKVCSREGRLAD-AVQIISYAVDKDLKWCILTGISTQD--------GGKTIDGSMQLYSMELKKQQQLEGHAACFNNIVIDEAVGPQPVICFTEKKRGSPD----FKLHIRDIYSS-RDG
P. falciparum   TKVFEKAQIFIDNNSQILYYSTDKDMKWCILCGISTQD--------QGKSIDGYMQLYSCEKKLHQTIEGFIGCFGSFVFDN-LDMKPLFCFVEKKKNSTI----SRLHLMDIYTN-KTE
P. berghei      TKVFEKAQIFIDNNSQILYYGTDKEMKWCILCGISTQD--------QGKSIDGYMQLYSCEKKLHQIIEGFIGCFGSFIFEN-WDTKPLFCFIEKKKNSSM----SRLHLMDIYNS-KTE
P. yoelii       TKVFEKAQIFIDNNSQILYYGTDKEMKWCILCGISTQD--------QGKSIDGHMQLYSCEKKLHQIIEGFIGCFGSFIFEN-WDTKPLFCFIEKKKNSSM----SRLHLMDIYNS-KTE
P. chabaudi     TKVFEKAQIFIDNNSQILYYGTDKEMKWCILCGISTQD--------QGKSIDGHMQLYSCEKKLHQIIEGFIGCFGSFIFDN-WDTKPLFCFIEKKKNSSM----SRLHLMDIYNS-KTE
P. knowlesi     TKVFEKAQVFIDNNSQILYYGTDKDMKWCILCGISTQD--------QGKSIDGYMQLYSCDKKLHQTIEGFIGCFGSFIFDN-WEMKPLFCFVEKKKNSSI----SRIHLMDIYTN-KTE
P. vivax        TKVFEKAQVFIDNNSQILYYATDKDMKWCILCGISTQD--------QGKSIDGHMQLYSCDKKLHQTIEGFIGCFGSFIFDN-WDIKPLFCFVEKKKNSTV----SRLHLMDIYTN-KTE
T. annulata     ------TGKLLDSNTKIVGYSSDITNKWCLVFGIYSND--------QGVSIDGVIQLYSVDKRQQQLFEGYAGTFTQLRTNNQTLKKSNLLIFCEHKKNTHN---IKLHLMDIGSLGQSN
B. bovis        ------SGKLAEPSTKLVDYASDAENKWCILTGVYSTD--------QGATVEGAIQLYSTERRQQQLLEGYAGTFGKLRISTESPDATGLLVFCEHKRGGQT---SKLHCMDVYSQRTEG
N. caninum      VKICSREGRLAD-AVQIISYAVDKDLKWCILTGISTQD--------GGKTIDGSMQLYSMELKKQQQLEGHAACFNNIVMDEAVGPQPVICFTEKKRGSPE----FKLHIRDIYSS-REG
C. muris        --IFERSGELAESSTQVVGYQCNSDQRWCMLMGLSPCTIPNQGSNSGGVSVKGQIQLFSAEKRQQQLLEGFSGSFGEIILDDFISAHPTSIVCFIEKKQDQLN--AKLHIMDISTSRAEG
C. parvum       --IFERAGKLAEQSTQIVGYQTDSGQRWCMLMGLCPVTNESTGS----ISVKGQLQLFSVEKRQQQLIEGFSGNFGELIVDDFVHS-PASIVCFVEKKQEHSN--ARLHVMDISGQRAG-
C. hominis      --IFERAGKLAEQSTQIVGYQTDSGQRWCMLMGLCPVTNESTGS----ISVKGQLQLFSVEKRQQQLIEGFSGNFGELIVDDFVHS-PASIVCFVEKKQEHSN--ARLHVMDISGQRAG-
T. thermophila  --AFERAGNLANPNCQIIGYKVNTQGNWAALYGISSP---------DQRQINGDIQLYKLDENSQQPLTGHCCCFGQ-AKIHN-DTHLSDIFAYYDRKPGD--TVAKILLRELSAP----
S. pombe        --MFDRHSSLNG--TQIISYKSNYNEEWFTLIGISS----------RDNRIAGNLQLYSKKRKVSQPLESHASAFAV-IQPEG-VDHEVQVLALASRLPTG----SKLSIVEVDRN--PN
S. cerevisiae   --LTLRHANLNN--TQIINFVANKNLDWFAVVGILQ----------ENGRIAGRIQLFSKQRNISQAIDGHVAIFTN-ILLEGNGSTPVQVFVTGNRNATTG--AGELRIIEIDHD--AS
C. merolae      --VFDRHPALAA--CQVINLVAGVGERWLALVGIRADDT-------APGGVLGKIQLYAVDRGVSQILDGYAAGFCDRYRIDSQDVTGTHLFAFASK--------TKFHVVEVAQDRKPD
D. discoideum   --VFDRHPDLQN--TEIINYRSDSTQNWLVLVAIHQ----------RDGRVVGRIQLYSVEKQISQSIEGHAACFAN-YIVPG-ATRPSTLFAISSRTQNA----SKILVLEVSKG--DG
A. thaliana     --MFDRTANLAN--NQIINYKCSPNEKWLVLIGIAPGSPE------RPQLVKGNMQLFSVDQQRSQALEAHAASFAQ-FKVPG-NENPSILISFASKSFNAGQITSKLHVIELGAQ--PG
D. melanogaster --MFDRHSSLNG--CQIINYRCNASQQWLLLVGISA----------LPSRVAGAMQLYSVERKVSQAIEGHAASFAT-FKIDA-NKEPTTLFCFAVRTATG----GKLHIIEVGAP--PN
H. sapiens      --MFDRHSSLAG--CQIINYRTDAKQKWLLLTGISA----------QQNRVVGAMQLYSVDRKVSQPIEGHAASFAQ-FKMEG-NAEESTLFCFAVRGQAG----GKLHIIEVGTP--PT

                       370       380       390       400       410       420       430       440       450       460       470       480
                ....|....|....|....|....|....|....|....|....|....|....|....|....|....|....|....|....|....|....|....|....|....|....|
T. gondii       G-QTPLRLAVDLRMPEDAPT--------------------DFPLSIHISQKFGVVYIVTKGGYLILLDALTGTELFRHRILQDAVFLATD---SPQTGGILTVNKRGLVCLCNINLQALI
P. falciparum   T-TIPYKIVKEINLINENLN--------------------DFPIYISLNTLQGVIYIVTKCSYVYVFDEGTLSLIVKEKISEDNIFICCD---SKNGEGIYAVNKKGIIFYITINYFHLI
P. berghei      G-STPYKIVKEINLINDTLN--------------------DFPIYISLNTLQGVIYVITKCSYVYIFDESTLTEIVREKISDDNIFICCD---NKNGEGIIAVNKKGKIYYITLNYINLI
P. yoelii       G-STPYKIVKEINLINDTLN--------------------DFPIYISLNTLQGVIYVITKCSYVYIFDESTLTEIVREKISDDNIFICCD---NKNGEGIIAVNKKGKIYYITLNYISLI
P. chabaudi     G-STPYKIVKEINLINDTLN--------------------DFPIYISINTLQGVIYVITKCSYVYIFDESTLTEIVREKISDDNIFICCD---NKNGEGVIAVNKKGKIYYITLNYISLI
P. knowlesi     TGTMPYKIVKEINLINEHIS--------------------DFPIYMSLNTLQGVIYIVTKCSYVYIFDEGTLTQLVKEKISEDNIFICCD---RKNGEGIYAVNKKGKIYYITINYVNLI
P. vivax        AGTMPYKIVKEINLINEHIS--------------------DFPIYISLNTLQGVIYIVTKCSYVYIFDEGTLTQLVKEKISEDNIFTCCD---SKNGEGIYAVNKKGKIYYITINYVNLI
T. annulata     TSNGVSSGVGTSVGTTGTTTGGTDILKINCIMERNEEFPNDFPISIHVFDSNGIILILTKNGFAHFYFSNTLTHLFTVRISMYSLFVSCNKKENNKSIGALTVNRIGEVINIIIDENNLL
B. bovis        SP--VPLKVSKDIEGLHSNTG-------------------DFPRFVHILDSCGFVCIITKCGFAYYHDVATATPLYSCKISDSPIFAAAAKYVDGKVAGSIAVNQSGDIIEILIDENRIL
N. caninum      G-QTPLRLAVDLQMPADAPT--------------------DFPLSIHISQKFGVVYIVTKGGYLMLLDALTGTELFRHRILQDAVFLATD---SPQTGGILTVNKRGLVCLCNINLQTLI
C. muris        STPFKMNVELPSNLNYNNPQVPGVH------GPGENSGIFDFPIYSYISNYFGVIFIITRGGILYVVEPSSGSILYFSQICQDSVFLGSP---SRS-HGICVVNRRGLVLHVMVNSNNIS
C. parvum       SGEFGSSLSPPTPFKAVIELPTVDE------GSAGGAHSFDFPIYTYVSSYFGVIFIITRGGILYIVEPTSNTLLYCNKVCQDSVFLGSP---SRF-HGICMANKKGLVLHITLNSSSVL
C. hominis      SGEFGSSLSPPTPFKAVIELPTVDE------GSAGGAHSFDFPIYTYVSSYFGVIFIITRGGILYIVEPTSNTLLYCNKVCQDSVFLGSP---SRF-HGICMANKKGLVLHITLNSSSVL
T. thermophila  -PEGFQKYKGAAEYPFNAQFP------------------ADFPIYMQVCTKYGQLFCLSKSGFLFVYELTTSALIYQQKVTDSSIFVATQ---DSKEDGIYAIAKNGNVILIQIDPSQLV
S. pombe        NP-AFATKTVDLFFPPEAVN--------------------DFPIAIEIGSTYNVAYVVTKYGFIHVYDLETAKCIYMNRVSGESIFVTTA---HKSVNGLMAINRKGQVLSVSINPETII
S. cerevisiae   LPSQYQKETTDIFFPPDATN--------------------DFPIAVQVSEKYGIIYLLTKYGFIHLYELETGTNLFVNRITAESVFTAAP---YNHENGIACINKKGQVLAVEISTSQIV
C. merolae      GAPRFEKRITNIVYAPGMDN--------------------DFPVALQVSSKLSVAYLLTKYGYIHAFDLESGKLIYANRISESPVFVSTS---HRETGGLIGINRRGQVLVFAIDPSLVV
D. discoideum   PN--FQKRASDVFYPPEIGAS-------------------DFPVAMQVSEKYEVIYMVTKLGYIHLFDLGTANLIYRNRISNENIFVTAF---EESTNGIIAVNRKGQVLSVSIDDKNII
A. thaliana     KP-SFTKKQADLFFPPDFAD--------------------DFPVAMQVSHKFNLIYVITKLGLLFVYDLETASAIYRNRISPDPIFLTSE---ASSVGGFYAINRRGQVLLATVNEATII
D. melanogaster GNQPFAKKAVDVFFPPEAQN--------------------DFPVAMQVSAKYDTIYLITKYGYIHLYDMETATCIYMNRISADTIFVTAP---HEASGGIIGVNRKGQVLSVTVDEEQII
H. sapiens      GNQPFPKKAVDVFFPPEAQN--------------------DFPVAMQISEKHDVVFLITKYGYIHLYDLETGTCIYMNRISGETIFVTAP---HEATAGIIGVNRKGQVLSVCVEEENII
```

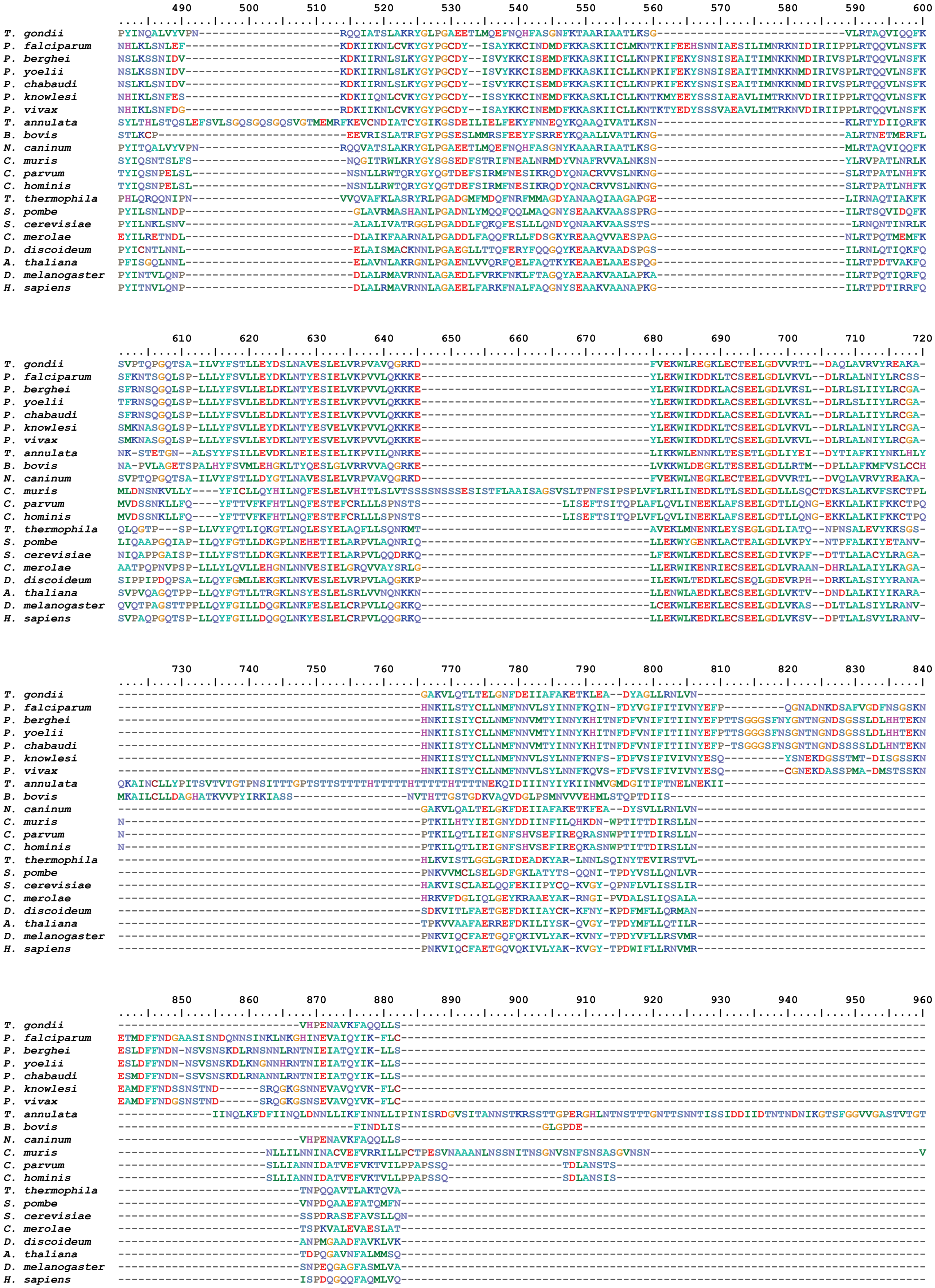
490 500 510 520 530 540 550 560 570 580 590 600
610 620 630 640 650 660 670 680 690 700 710 720
730 740 750 760 770 780 790 800 810 820 830 840
850 860 870 880 890 900 910 920 930 940 950 960
T. gondii
P. falciparum
P. berghei
P. yoelii
P. chabaudi
P. knowlesi
P. vivax
T. annulata
B. bovis
N. caninum
C. muris
C. parvum
C. hominis
T. thermophila
S. pombe
S. cerevisiae
C. merolae
D. discoideum
A. thaliana
D. melanogaster
H. sapiens

```
                        970       980       990      1000      1010      1020      1030      1040      1050      1060      1070      1080
                ....|....|....|....|....|....|....|....|....|....|....|....|....|....|....|....|....|....|....|....|....|....|....|....|
T. gondii       ---SEPPLADVTQVSEVLLQQHKYQEFTSLMLDFLKGNKPEQGPLQTRLLEVNLLH-SPQVAETIFQMEMLTHFDRAKIAALCEKVGLSQRALELYTDIADIKRVMLQSG----------
P. falciparum   ---ENNISFDINKIIDYLLSKKKLQEATSILLDYLKENKAEHKNLQTKLFEFNLYN-NVQVAETLFQMDIFTYYDKNKIAYLCEEKGLYQRALENYTNINDIKRVITKSTCFQKGGNNNN
P. berghei      ---DNNIQLDIGKIVDYLLNNNKLQEATSILLDYLKDNKPEHKHLQTKIFEFNLYH-NVQIAETIFQMDIFTYYDKNRIAYLCEEKGLYQRALENYTNINDIKRVITKSACFQKNGNISN
P. yoelii       ---DNNIQLDIGKIVDYLLNNNKLQEATSILLDYLKDNKPEHKHLQTKIFEFNLHH-NVQIAETIFQMDIFTYYDKNRIAYLCEEKGLYQRALENYTNINDIKRVITKSACFQKNGNISN
P. chabaudi     ---DNNIQLDIGKIVDYLLNNNKLQEATSILLDYLKDNKPEHKHLQTKIFEFNLYH-NVQIAETIFQMDIFTYYDKNRIAYLCEEKGLYQRALENYTNINDIKRVITKSACFQKNGNISN
P. knowlesi     ---ENNIPFDINNIIDYLLSKKKLQEATSILLDFLKDNKPEHKNLQTKLFEFNLYN-NVQVAETLFQMDIFTYYDKNRIAYLCEEKGLYQRALENYTNLNDIKRVITKSSCFQKNTNMNH
P. vivax        ---ENNIPFDINNIVDYLLSKKKLQEATSILLDFLKDNKPEHKNLQTKLFEFNLYN-NVQVAETLFQMDIFTYYDKNRIAYLCEEKGLFQRALENYTNLNDIKRVITKSSCFQKSTNSSQ
T. annulata     VTVENVLLCNIIEIMEKLIEMKKLKEMNEILLDYLKEDLEIHEKLQTRLLQVNIEN-DKRIGEEILKLDILTKFNKNYIAKLCEDNELYEYSLKYYNDITNIKRILIKGIY---------
B. bovis        ------PLCDVGSVAEILIKHNKLQELTKILLEYLKPNRVEHAALQTRLLEVNLQQ-QPRVADMILQLNVLTHFDRAYIARLCEDAGMFDMAIQHYNSFFDVKRLIIKAGG---------
N. caninum      ---AEPPLADVNQVIDVLLQQRKFQEFTSLLLDYLKSNKPEQGPLQTRLLEVNLLH-SPQVAETIFQMDMLTHFDRAKVAALCEKAGLSQRALEFYTDIADIKRVMLQSG----------
C. muris        LPHGLELAIDKISIVDIFISHGHYKEITSILLDYLKNNRPEDAALQTKLFELNLLY-APQVAEALFQMDVYTYYDKHAIASLCEKAGLFERCLENYTDMRDIRRILSISCG---------
C. parvum       -AESLDLNIDKTSVVEIFVSHSRYKEITSILLDHLKANKPEDSALQTKLLEVNLLH-APQVAEALFQMDLFTHYDKHAIAALCEKAGLYERALENFSDMRDIRRILGVACG---------
C. hominis      -AESLDLNIDKTSVVEIFVSHSRYKEITSILLDHLKANNPEDSVLQTKLLEVNLLH-APQVAEALFQMDLFTHYDKHAIAALCEKAGLYERALENFSDMRDIRRILGVACG---------
T. thermophila  ----TNPSTNVHQIAEIFGQAGKFKEMSAFLVDCMKQNRPEDGPWQTKVLEWNIIN-EPNIVDPIFDLTKWNQYNRLRIAQLCEQKQLYQRALENYSDIKDIRRVCLNT-----------
S. pombe        ----SNPSINLEKIVDIFMSQNLVQQATAFLLDALKDDNPEHSHLQTRLLEINLIN-APQVADAILGNQMFTHFDRAVIASLCERAGLVQRALELYDKPADIKRVIVHS-----------
S. cerevisiae   --PETASQIDIEKIADLFFSQNHIQQGTSLLLDALKGDTPDQGHLQTRVLEVNLLH-APQVADAILGNNIFSHYDKPTIASLSEKAGLYQRALENYTDIKDIKRCVVHT-----------
C. merolae      ------DTADHAAVADLFLQRGMVQEATSYLLDVMREDRPEQGDLQTKVLEINLTLGAPQVADAILAQEMYTHFDKQRIAVLCERAGLAQRALQLYTRLSDVKRILVTLS----------
D. discoideum   --EEGGPYIDANQVVELFSARNMIQETSNFLFAILDGDRPQDANLQTKLLEINLLH-APQNADAIMGGQKFTHYNRLRIGGLCEKAGLYQRALEHYTDLADIKRVLSHAG----------
A. thaliana     --MEGGCPVDYNTITDLFLQRNLIREATAFLLDVLKPNLPEHAFLQTKVLEINLVT-FPNVADAILANGMFSHYDRPRVAQLCEKAGLYIQSLKHYSELPDIKRVIVNT-----------
D. melanogaster --EEE-PLADINQIVDIFMEHSMVQQCTAFLLDALKHNRPAEGALQTRLLEMNLMS-APQVADAILGNAMFTHYDRAHIAQLCEKAGLLQRALEHYTDLYDIKRAVVHT-----------
H. sapiens      --DEE-PLADITQIVDVFMEYNLIQQCTAFLLDALKNNRPSEGPLQTRLLEMNLMH-APQVADAILGNQMFTHYDRAHIAQLCEKAGLLQRALEHFTDLYDIKRAVVHT-----------

                       1090      1100      1110      1120      1130      1140      1150      1160      1170      1180      1190      1200
                ....|....|....|....|....|....|....|....|....|....|....|....|....|....|....|....|....|....|....|....|....|....|....|
T. gondii       -----------------------GKISQEFTQQFFGNLPPDASLEILTDML-------------------------------------------RSS-SQNLQAVVAVAIKFHGQIG---
P. falciparum   TTTTTTTTSNMGDGHFDMNNVSKGKISIEWIKNYFSTLSDSVCEELLFDFM-------------------------------------------KGS-KINMEVVISICVQYYDKIG---
P. berghei      NLNSEGGSGNMS---------LHGGISLEWIKNYFSTLSDSVCQDLLFDFM-------------------------------------------KGN-KVNIEIIISICVQYYNKIG---
P. yoelii       NLNSEGGSGNMS---------LHGGISLEWIKNYFSTLSDSVCQDLLFDFM-------------------------------------------KGN-KVNIEIIISICVQYYNKIG---
P. chabaudi     NFNSEGGSGNMS---------LHGGISLEWIKNYFSTLSDSVCQDLLFDFM-------------------------------------------KGN-KVNIEIIISICVQYYNKIG---
P. knowlesi     NMEDSLPNR---------------AISIDWIKKYFSTLSDSVCQDILFDFM-------------------------------------------KGN-KINMEVVISICVQYYNKIG---
P. vivax        -MDGSLPNR---------------GISIDWIKKYFSTLSDSVCQDILFDFM-------------------------------------------KGN-KINMEVVISICVQYYNKIG---
T. annulata     ------------------------FLSQNIINQTLLTFNIQDSLYFLQQLH--------------------------------------------NEYIGISGGVGDVSREFEGEFGGEF
B. bovis        ------------------------NMNRAVLEKSMKNMSPENALEVLREML--------------------------------------------DSAEISNDHVVSCALTMHNHIG---
N. caninum      -----------------------GKISQEFTQQFFGNLPPEASLEILTDML-------------------------------------------RSS-SQNLQAVVAVAIKFHGQIG---
C. muris        ------------------------SLNSDWLANYLSKLPSNTRFECLKELLSVCRNQ---GGNINSGSGIG---LMSTN-----TSDSSGTSLTNTINNSILQNLIQVCIKNIDNIG---
C. parvum       ------------------------SLNTDWLANYLSKLSPRTRFDCLKELLLVCKNSSGAGGLVSMGGGLASNGALPGNGQAGLIGNSSSNSIALSNNNTVLQSVIQVCIKNVDNIG---
C. hominis      ------------------------SLNTDWLANYLSKLSPRTRFDCLKELLLVCKNSSGAGGLVSMGGGLASNGALPGNGQAGLIGNSSSNSIALSNNNTVLQSVIQVCIKNVDNIG---
T. thermophila  -----------------------HMIQPAWLVNYFSNVQTDWALACMQDLL-------------------------------------------KHN-RQNLPLVVEACAKNYQKFT---
S. pombe        -----------------------NLLNPEWLMNYFSRFSPDEVYDYLREML-------------------------------------------RSNLRQNLQIVVQIATRYSDLVG---
S. cerevisiae   -----------------------NALPIDWLVGYFGKLNVEQSLACLKALM-------------------------------------------DNNIQANIQTVVQVATKFSDLIG---
C. merolae      -----------------------HAIQPEYFLSIFANLSPDDSLELLDELL-------------------------------------------TNDLRGNIQRVVQVAIRYSDALG---
D. discoideum   -----------------------HMVNQEFLVSYFGSLNPEDRMECMRDFL-------------------------------------------RTNPRQNLQLVVAIAVSYSDQIT---
A. thaliana     -----------------------HAIEPQALVEFFGTLSSEWAMECMKDLL-------------------------------------------LVNLRGNLQIIVQACKEYCEQLG---
D. melanogaster -----------------------HMLNAEWLVSFFGTLSVEDSLECLKAML-------------------------------------------TANLRQNLQICVQIATKYHEQLT---
H. sapiens      -----------------------HLLNPEWLVNYFGSLSVEDSLECLRAML-------------------------------------------SANIRQNLQICVQVASKYHEQLS---

                       1210      1220      1230      1240      1250      1260      1270      1280      1290      1300      1310      1320
                ....|....|....|....|....|....|....|....|....|....|....|....|....|....|....|....|....|....|....|....|....|....|....|
T. gondii       ---------------------TTKLVEMFEKFSSYEGVFYFLGSILAFSSDPE-------------------------------------------------------------------
P. falciparum   ---------------------IKKIINKFEENKNYEGIFYFVSNILNDLPNLIVKGNN------NDNLSN-------NSLSTYNNMNDEDASILLTSDICSTPQYSY-ETANLKLEDVHY
P. berghei      ---------------------IKKIVNKFEENKNYEGIFYFISSILNELQNMSTARNANDIGKYNDNNSISSNNNINNNLSSSRSNNDDASSILLNSDIGIGTQFSYENNSALTIEDLHH
P. yoelii       ---------------------IKKIVNKFEENKNYEGIFYFISSILNELQNMGTVGNANDIGKYNDNNSISSNNNINNNLSSSRSNNDDTSSILINSDIGIGTQFPYENNSVLTIEDLHH
P. chabaudi     ---------------------IKKIVNKFEENKNYEGIFYFISSILNELQNMGTVGNANDMGKYNDNNSISSNNNINNNLSSSRSNNDDASSILLNSDIGIGTQFSYENNSVLTIEDLHH
P. knowlesi     ---------------------IKKIINKFEENKNYEGIFYFVSSILTHLPNLTSKST--------DHLS-------------YGNMNDDASSILLASDIGSQYSNAE-NSSNLKIEDVHF
P. vivax        ---------------------IKKIINKFEENKNYEGIFYFVSSILTHLPNLTNKAT--------DHLS-------------YGSMNDETSSILLASDIGSQYNNAE-NPSSLKIEDVHF
T. annulata     EEKFDEKNRKVEEKVDRLENIIINICLILYNKINLKELTNILTNKLFYKFFKKLSKQE-------------------------------------------------------LDEDINE
B. bovis        ---------------------TMQVVQLFERSASSDVLFSFLR---ALPVISQGSAAE-------------------------------------------------------STEQNAT
N. caninum      ---------------------TTKLVEMFEKFSSFEGIFYFLGSILAFSTDPE-------------------------------------------------------------------
C. muris        ---------------------IENIITLFEQQNLWEGIYYVIGSNLTAYLGGVDSATSG--------------------IIGNTTT----GPSISTNNLNSHLNNKSTSIQQSFSSQS-F
C. parvum       ---------------------IENIITLFEQQGIWEGIYYVVGSNLTQYLSGNSLSSSGSTDSLNLSSES---------LIGDSGTQNRGGAGVVLAGSGRLPSSNTGSVNGGTGSFSTF
C. hominis      ---------------------IENIITLFEQQGIWEGIYYVVGSNLTQYLSGNSLSSSSSAESLNLSSES---------LIGDSGTQNRGGAGVVLAGSGRLPSSNTGSVNGGTGSFSTF
T. thermophila  ---------------------VQSCIKCFESVGSFDGVYTFLSQIINSTEDKD-------------------------------------------------------------------
S. pombe        ---------------------AQRIIEMFEKFKTFEGLYYYLGSIVNITEDPE-------------------------------------------------------------------
S. cerevisiae   ---------------------PSTLIKLFEDYNATEGLYYYLASLVNLTEDKD-------------------------------------------------------------------
C. merolae      ---------------------PSRLIDLFEKHQCYEGMYLYLGAILATSKSPY-------------------------------------------------------------------
D. discoideum   ---------------------PEAIIAMFESFRLYEGLYLYLTQVVVTSTSPE-------------------------------------------------------------------
A. thaliana     ---------------------VDACIKLFEQFKSYEGLYFFLGSYLSMSEDPE-------------------------------------------------------------------
D. melanogaster ---------------------NKALIDLFEGFKSYDGLFYFLSSIVNFSQDPE-------------------------------------------------------------------
H. sapiens      ---------------------TQSLIELFESFKSFEGLFYFLGSIVNFSQDPD-------------------------------------------------------------------

                       1330      1340      1350      1360      1370      1380      1390      1400      1410      1420      1430      1440
                ....|....|....|....|....|....|....|....|....|....|....|....|....|....|....|....|....|....|....|....|....|....|....|
T. gondii       VHFKYIEAAAKLNHTQEVERVCRE-SKCYEPQRVKEFLKQVKLPDPRPLIYVCDLHGYVTELAEYLFK-----------NSLLKYIEVYVSRVNSA-------NAPLVIGTLIDQDAA--
P. falciparum   IMFKYIEACVKINNIQELDRICKDKNAKYNPEQIKNFLKDCKLSDPRPLIYVCDIHNYIEELAEYLYK-----------NSLLKYIEVYVIKVNPN-------NAHKVIGVLLDLDAS--
P. berghei      IMFKYIEACVKINNIQELDRICKDRNAKYNPEQIKNFLKECKLSDPRPLIYVCDIHNFIEELAEYLYK-----------NSLLKYIEVYVIKVNPH-------NAHKVIGVLLDLDAS--
P. yoelii       IMFKYIEACVKINNIQELDRICKDRNAKYNPEQIKNFLKECKLSDPRPLIYVCDIHNFIEELAEYLYK-----------NSLLKYIEVYVIKVNPH-------NAHKVIGVLLDLDAS--
P. chabaudi     IMFKYIEACVKINNIQELDRICKDRNAKYNPEQIKNFLKECKLSDPRPLIYVCDIHNFIEELAEYLYK-----------NSLLKYIEVYVIKVNPH-------NAHKVIGVLLDLDAS--
P. knowlesi     IMFKYIEACVKINNIQELDRICKDKNAMYNPEQIKNFLKDCKLSDPRPLIYVCDIHNYIEELAEYLYK-----------NSLLKYIEVYVIKVNPN-------NAHKVIGVLLDLDAS--
P. vivax        IMFKYIEACVKINNIQELDRICKDKNAKYNPEQIKNFLKDCKLSDPRPLIYVCDIHNYIEELAEYLYK-----------NSLLKYIEVYVIKVNPN-------NAHKVIGVLLDLDAS--
T. annulata     IIIRYMKLCIEYNDIIELENILLY-NNNFNLFDAKELIKNSQLSTTKPFIIICYRLNNIQELLLFLFNYYDTVLAQPDTNSTNNLTTSTIGTVVPGIKDTTTVGASTVTGTVKEELILKD
B. bovis        IVYTFIKCCIDRNEMEDLERICKE-SNVYDGVRVKDLLKQSALPNPKSLLIVCHKLGELAELTEYLY-----------RNGMEKAIEVYVNTINPG-------GVATVVSTLFD------
N. caninum      VHFKYIEAAAKLNHTQEVERVCRE-SKCYEPQRVKEFLKQAKLPDPRPLIYVCDLHGYVTELAEYLFK-----------NSLLKYIEVYVSRVNSA-------NAPLVIGTLIDQDAA--
C. muris        IIFKYIEASINLGQVQEAERICRDFPQSYDPDQVIEYFKNIKMNDLRPLIWVCDLHHRVEDLINYLYH-----------MSLYKYIQVYALKINPS-------QTPLVIGTLIDLDGS--
C. parvum       IVFKYIEASVHLGQIQEAERICRDFPQSYEPEQVIEYFKSIKMSDLRPLIWVCDLHHRVEELISYLYH-----------MSLYKYIQVYTLKINPS-------QTPLVIGTLIDLDGS--
C. hominis      IVFKYIEASVHLGQIQEAERICRDFPQSYEPEQVIEYFKSIKMSDLRPLIWVCDLHHRVEELISYLYH-----------MSLYKYIQVYTLKINPS-------QTPLVIGTLIDLDGS--
T. thermophila  IHFKYIEAATKCNQLRIVEQVIQEKANCYDPVKVKDFLKGLRLQDPKPLIFLCDIHKYIDELTAYLYQ-----------NNFLNYIQIYLFQVNPQ-------AAPIVLGTLIDQECE--
S. pombe        VVYKYIQAACLMNQFTEVERICRD-NNVYNPEKVKNLLKEAKLADQLPLILVCDRYDFVNDLVFYLFR-----------NNMFQFIEIYVQRINPS-------KTPQVVGALLDIDCD--
S. cerevisiae   VVYKYIEAAAKMKQYREIERIVKD-NNVYDPERVKNFLKDANLEDQLPLVIVCDRFDFVHEMILYLYK-----------SQNLKFIETYVQQVNPS-------KTAQVVGALLDMDCD--
C. merolae      VVAKYIEAACRTQQWSEAERVTRE-SDLYNPEQVKELLFELRPKDPRPIINVCEKGGDVEDMVRYFVK-----------QNQLEIVKGYVQRINPG-------NTPAVVAALLDSDSSS-
D. discoideum   VHFKYIEAAAKINQFKEVERMCRD-SNYYDPEKTRDFLKEAKLPDQLPLIIVCDRYEFISDLTNYLYK-----------NNLSKYIEAYVQKINPV-------NTPLVVGALLDLDCQ--
A. thaliana     IHFKYIEAAAKTGQIKEVERVTRE-SNFYDAEKTKNFLMEAKLPDARPLINVCDRFGFVPDLTHYLYT-----------NNMLRYIEGYVQKVNPG-------NAPLVVGQLLDDECP--
D. melanogaster VHFKYIQAACKTNQIKEVERICRE-SNCYNPERVKNFLKEAKLTDQLPLIIVCDRFDFVHDLVLYLYR-----------NNLQKYIEIYVQKVNPS-------RLPVVVGGLLDVDCS--
H. sapiens      VHFKYIQAACKTGQIKEVERICRE-SNCYDPERVKNFLKEAKLTDQLPLIIVCDRFDFVHDLVLYLYR-----------NNLQKYIEIYVQKVNPS-------RLPVVIGGLLDVDCS--
```

```
                  1450      1460      1470      1480      1490      1500      1510      1520      1530      1540      1550      1560
              ....|....|....|....|....|....|....|....|....|....|....|....|....|....|....|....|....|....|....|....|....|....|....|....|
T. gondii       -----------------------EDFIRNLLQSVRGGCS---AQQLVEEFEKRNRLRLLLQWLEARVAEGNQEPAVHNALAKIYIDTNRDAESFLKTNAYYDSLVVGKYCEERDPHLAFT
P. falciparum   -----------------------EDFLLNLLNNIKNISN---IGNLIEIAEKRNRLKLLLPWLESRANEGYENIELHNALAKIYIDLNKDPENFLKNNNFYDKKMIGKYCEDLDPHLAYT
P. berghei      -----------------------EDFLLNLLNNIKNISN---IGNLIDIAEKRNRLKLLLPWLESRSNEGYENIELHNALAKIYIDLNKDPETFLKNNNFYDKKMIGKYCEDLDPHLAYT
P. yoelii       -----------------------EDFLLNLLNNIKNISN---IGNLIDIAEKRNRLKLLLPWLESRSNEGYENIELHNALAKIYIDLNKDPETFLKNNNFYDKKMIGKYCEDLDPHLAYT
P. chabaudi     -----------------------EDFLLNLLNNIKNISN---IGNLIDIAEKRNRLKLLLPWLESRSNEGYENIELHNALAKIYIDLNKDPETFLKNNNFYDKKMIGKYCEDLDPHLAYT
P. knowlesi     -----------------------EDFLLNLLNNIKNISN---IGNLIEIAEKRNRLKLLLPWLESRSNEGYENIELHNALAKIYIDLNKDPENFLKNNNFYDKKLIGKYCEDLDPHLAYT
P. vivax        -----------------------EDFLLNLLNNIKNISN---IGNLIEIAEKRNRLKLLLPWLESRSNEGYENIELHNALAKIYIDLNKDPENFLKNNNFYDKKLIGKYCEDLDPHLAYT
T. annulata     IKILLEMNPKLLTEVIIILLELSEELINKILINITNITL---LKEIIKLIEERHQLIILKPFLEMKCKEIN-EPILHTALMKIYITLGENPEDYLINNKYYLKEEIANYCQDIDLQLSLL
B. bovis        -------------------LSASEHVIHSILENLHDPNG---MKALIQIADERHQLLMLRDWLEKRVEEGHKETEIHTALAKIRVSSQKDAEQFLSTNKIYDRSIIGRFCEDRDPMLAYL
N. caninum      -----------------------EDFIKNLLQSVRGGCS---AQQLVDEFEKRNRLRLLLQWLEARVAEGNQEPAVHNALAKIYIDTNRDAENFLKTNAYYDSLVVGKYCEERDPHLAFT
C. muris        -----------------------EDLVKSLLQEIKTLGSSFSFSDLIQQAEKRNRLKLLLPWLEERVHEGYQDPSLHNALAKIYIDMNRDPENFLKTNPYYDSKLIGDYCEDRDPYLAYI
C. parvum       -----------------------EDLVKSLLQEIKTLGSSFSFGELIQQAENRNRLKLLLSWLEERVQEGYQDPALHNALAKIYIDMNKDSENFLKTNPHYDAKTIGDYCEDRDPYLAYI
C. hominis      -----------------------EDLVKSLLQEIKTLGSSFSFGELIQQAENRNRLKLLLSWLEERVQEGYQDPALHNALAKIYIDMNKDSENFLKTNPHYDAKTIGDYCEDRDPYLAYI
T. thermophila  -----------------------ERQIQIILQQIAGKLIPQCYEPLIEEFEKRNRLKRVLESWLEGRCNEGNQIPAIHNALAKIKIDTNQDPETFLQNNQFYDAKVVGKFCEERDPHLAVI
S. pombe        -----------------------EELVQNLLMSVVGQVP---VDELVEEVERRNRLKLLLPYLESLLQSGSQDRAIYDALAKIYIDSNNNPEVFLKENNFYDTLTVGKYCEKRDPYLAFI
S. cerevisiae   -----------------------EAFIQSLLQSVLGQVP---INELTTEVEKRNRLKILLPFLEQSLSQGIQDQAVYNALAKIYIDSNNSPEKFLKENDQYDTLDVGHYCEKRDPYLAYI
C. merolae      ----------------------VDDVYIKSLLMSIKSLVP---IEAVTQVLEERQKLKMLQLLEALVAEGSKDRAVHTALAKVYVDANINPEHFLETNEFYDARIVGNFCERRDPLLAFI
D. discoideum   -----------------------EDYLRNLIMSVRNMCP---ADSLVEQVEKRNRLKLLLPWLEARVAEGNIEPAIHNALAKIYIDSNKNPEAFLLHNQFYDSKVVGKYCEKRDPYLSFV
A. thaliana     -----------------------EDFIKGLILSVRSLLP---VEPLVAECEKRNRLRLLTQFLEHLVSEGSQDVHVHNALGKIIIDSNNNPEHFLTTNPYYDSKVVGKYCEKRDPTLAVV
D. melanogaster -----------------------EDIIKNLILVVKGQFS---TDELVEEVEKRNRLKLLLPWLESRVHEGCVEPATHNALAKIYIDSNNNPERYLKENQYYDSRVVGRYCEKRDPHLACV
H. sapiens      -----------------------EDVIKNLILVVRGQFS---TDELVAEVEKRNRLKLLLPWLEARIHEGCEEPATHNALAKIYIDSNNNPERFLRENPYYDSRVVGKYCEKRDPHLACV
```

```
                  1570      1580      1590      1600      1610      1620      1630      1640      1650      1660      1670      1680
              ....|....|....|....|....|....|....|....|....|....|....|....|....|....|....|....|....|....|....|....|....|....|....|....|
T. gondii       AYKRAWGACDEQLVELTNKNGLFRLQARYLVERQAPELWAYVLREENPH-------------RRQAIDQVVSSALPESSS-ADEVSAAVNAFINAQLPHELIELLEKIVLHNS--DFSNN
P. falciparum   AYERSNGECDEELIYITSKNGLFKLQARYLISRQSIELWKVVLDETNKY-------------RKNVIDQVIGSTLIESNN-ADEITVTVKAFIEKKLSSELIELLEKIVLHNS--EFSDN
P. berghei      AYERSNGQCDEELINITSKNGLFKLQAKYLVSRQSMGLWSMVLDELNKY-------------RKNVIDQVIGSTLIESNN-ADEITVTVKAFIEKKLSSELIELLEKIVLHNS--EFRDN
P. yoelii       AYERSNGQCDEELINITSKNGLFKLQAKYLVSRQSMGLWSMVLDESNKY-------------RKNVIDQVIGSTLIESNN-ADEITVTVKAFIEKKLSSELIELLEKIVLHNS--EFRDN
P. chabaudi     AYERSNGQCDEELINITSKNGLFKLQAKYLVSRQSMGLWSMVLDESNKY-------------RKNVIDQVIGSTLIESNN-ADEITVTVKAFIEKKLSSELIELLEKIVLHNS--EFRDN
P. knowlesi     VYEKSNGECDEELIHITSKNGLFKLQAKYLVSRQSMELWIKVLDESNKY-------------RKNVIDQVIGSTLIESNN-AEEITVTVKAFIEKKLSSELIELLEKIVLHNS--EFSDN
P. vivax        VYEKSNGECDEELINITSKNGLFKLQAKYLVSRQSMELWKRVLDESNKY-------------RKNVIDQVIGSTLIESNN-AEEITVTVKAFIEKKLSSELIELLEKIVLHNS--EFSDN
T. annulata     IYKHN--SMYEHFIKFSLKHQLYKSLYQFLLKESNIKFYQYYYSNCNDMK-------------------EIIKLCIENCN-SIEISVLIKFLLSENLNEDLIILLEGLLLNQT--EFTNN
B. bovis        VYSEA--QLDSDVLRLCIGNGFYKMLAAYALKRSSPSLWHDIFSETSGLDDD-----------NRKHVCEELVILAPDSSN-ASEISCALKALLDAGMNEEVIALLEQLLLKQT--QFSSS
N. caninum      AYKRAWGACDEQLVDLTNKNGLFRLQARYLVERQAPELWAYVLREENPH-------------RRQAIDQVVSSALPESSS-ADEVSAAVNAFINAQLPHELIELLEKIVLHNS--DFSNN
C. muris        AYRKAWGLCDDEIIQVTYKNDLYRLLARYLVERQDLSLWSKVLGSGNKQSEDLEGDEHFDSSRQLIIDQVISSILPEFSNKAEEVSCTIRAFINADIPTSLLELLEKIVLHINNAEFTQN
C. parvum       AYRKAWGQCDDEIIQVTYKNDLYRLLARYLVERQDLDLWNKVLGNSSNSIEPVVN----EQCRQAIIDQVTSSILPEFYNKSEEISCVIRAFINAEVPNSLLEVLEKIIFHVNNTEFSQN
C. hominis      AYRKAWGQCDDEIIQVTYKNDLYRLLARYLVERQDLDLWNKVLGNSSNSIEPVVN----EQCRQAIIDQVTSSILPEFYNKSEEISCVIRAFINAEVPNSLLEVLEKIIFHVNNTEFSQN
T. thermophila  AYKRSWGQCDEELIMVTNKNALFRLQARYLVERQSKELWASVLTPDNPY-------------RKQVIEQVVQSALPESKD-VDQVSTAVKAFMDAELPEHLITLLEKIVLHNH--EFGQY
S. pombe        AYEK--GGNDTEIINLCNENSMFKQLARYLLKRSDSNLWSEVL-QDSAY-------------RRPLLDQVIATAVPESSD-PEAVSIVVKALMEVDLPSQLIELLEKIVLQPS--SFSEN
S. cerevisiae   AYEK--GQNDDDLIRITNENSMYKYQARYLLERSDLDLWNKVLNQENIH-------------RRQLIDSVISVGIPELTD-PEPVSLTVQAFMTNGLKLELIELLEKIILEPS--PFNEN
C. merolae      AYAH--GACDDDVLRVTSDHGLWREQARYVIERMNPELWARVLEESNPN-------------RKTLLTQVIGSALMENRN-PEQVSAAVKALMRAGLQNELLELLEKLVLQAGG-TFANN
D. discoideum   AYKR--GLCDYELIEVTNKNTLFKNQARYLVERQDPDLWAYVLSDQNEY-------------KRSLIDQVVQTALPESTN-ATEVSATVKAFMDANLPNELIELLEKIVLEGK--EFKTA
A. thaliana     AYRR--GQCDEELINVTNKNSLFKLQARYVVERMDGDLWEKVLTEENEY-------------RRQLIDQVVSTALPESKS-PEQVSAAVKAFMTADLPHELIELLEKIVLQNS--AFSGN
D. melanogaster AYER--GLCDRELIAVCNENSLFKSEARYLVGRRDAELWAEVLSESNPY-------------KRQLIDQVVQTALSETQD-PDDISVTVKAFMTADLPNELIELLEKIILDSS--VFSDH
H. sapiens      AYER--GQCDLELINVCNENSLFKSLSRYLVRRKDPELWGSVLLESNPY-------------RRPLIDQVVQTALSETQD-PEEVSVTVKAFMTADLPNELIELLEKIVLDNS--VFSEH
```

```
                  1690      1700      1710      1720      1730      1740      1750      1760      1770      1780      1790      1800
              ....|....|....|....|....|....|....|....|....|....|....|....|....|....|....|....|....|....|....|....|....|....|....|....|
T. gondii       KNLQNLLILTAMKAD---------------------------------------------------------SSRIIDYVNRLHNYDGAAIAQVAMEY--GLREEAFTIYKRFGLFGEAA
P. falciparum   KNLQNLLILTAIKSD---------------------------------------------------------SKKVMEYINRLDNFSGPQIAAVAYEY--KLREEAFVIYKKFNCYTSAI
P. berghei      KNLQNLLILTAIKSD---------------------------------------------------------SKKVMEYINRLDNFSGSQIAEVAYEY--KLREEAFVIYKKFNCNTSAI
P. yoelii       KNLQNLLILTAIKSD---------------------------------------------------------SKKVMEYINRLDNFSGPQIAEVAYEY--KLREEAFVIYKKFNCNTSAI
P. chabaudi     KNLQNLLILTAIKSD---------------------------------------------------------SKKVMEYINRLDNFSGPQIAAVAYEY--KLREEAFVIYKKFNCNTSAI
P. knowlesi     KNLQNLLILTAIKSD---------------------------------------------------------SKKVMEYINRLDNFSAPQIASVAYQY--KLREEAFVIYKKFNYYKLAI
P. vivax        KNLQNLLILTAIKSD---------------------------------------------------------SKKVMEYINRLDNFSGPQIASVAYDY--KLREEAFVIYKKFNCYTSAI
T. annulata     SNLQNLLLATTIKTYSNQLDDLNQLDYYSRSDSNKLDSNKLDDSIRLDSTKLDDSTKFSDSTKLNDPNQLDGPGKLEYYLNKLNNYDVNSLAKLANDLK--LHYSAFLIYNKSNKYNEAF
B. bovis        SNLQNLLLATAVKTN---------------------------------------------------------PSKLEEYLSKLDNYDVAALSKLSDSLG--QSRSSFAILKSAGRSLDAL
N. caninum      KNLQNLLILTAMKAD---------------------------------------------------------SSRIIDYVNRLQNYDGAAVAQVAMDY--GLREEAFTIYKKFGLFGEAA
C. muris        KNLQNLLLLTAIKVD---------------------------------------------------------PSRLDDYILRLDNYDAKEVAKIAIQHN--ILRSAFQIYKKFSLNIEAM
C. parvum       KNLQNLLLLTSIKID---------------------------------------------------------VRRLDDYVLRLDNYDAKEVAKVAIDHG--LYSHAFQIYKKFSFNNEAV
C. hominis      KNLQNLLLLTSIKID---------------------------------------------------------VRRLDDYVLRLDNYDAKEVAKVAIDHG--LYSHAFQIYKKFSFNNEAV
T. thermophila  KKLQNLLIITAIKSD---------------------------------------------------------KTKVMDYINRLDNYDGPEIAKIALGDPYQLYEEAYIIYVKFNQPVSAI
S. pombe        ANLQNLLFLTAIKAD---------------------------------------------------------KSRVMEYIDKLDKYDVDEIAEIAIEN--GLYEEAFRIYKIHNKHEQAM
S. cerevisiae   VALQGLLLLSAIKYE---------------------------------------------------------PTKVSSYIEKLDNYDADEIAPLCIEH--DLKEEAFEIYDKHEMYGKAL
C. merolae      TSLQNLLLLTSIRSA---------------------------------------------------------PERTGDYIRRLDAFDGATIGQLCLEH--DLYDEAFATFAKFEMHVEAV
D. discoideum   KELQNLLILTAIRAD---------------------------------------------------------KSRVTDYINRLDNFDGSKLAPIAIES--QLYEEAFFMYKKFQFNVEAI
A. thaliana     FNLQNLLILTAIKAD---------------------------------------------------------PSRVMDYINRLDNFDGPAVGEVAVDA--QLYEEAFAIFKKFNLNVQAV
D. melanogaster RNLQNLLILTAIKAD---------------------------------------------------------RTRVMDYINRLENYDAPDIANIAISN--QLYEEAFAIFKKFDVNTSAI
H. sapiens      RNLQNLLILTAIKAD---------------------------------------------------------RTRVMEYINRLDNYDAPDIANIAISN--ELFEEAFAIFRKFDVNTSAV
```

```
                  1810      1820      1830      1840      1850      1860      1870      1880      1890      1900      1910      1920
              ....|....|....|....|....|....|....|....|....|....|....|....|....|....|....|....|....|....|....|....|....|....|....|....|
T. gondii       DTLLK-------------------------------------------------------------------------------------------------------------------
P. falciparum   SVLLDKILYNKNQKNKNFHEFSSHDSTVPYYSNNNNKDNIFGNKSDNENDEYLKQFTQNNSYREKYGS-YYNNNDISNSNNNNIHLHPEFNNEDKFSSDTLHNYED-------IDSTEST
P. berghei      SVLLDNILIFNQNRKGKMSANARRKKTEFALFN------TINDYSVNDSDGYLNVTNFSGALSSSYNEKRDGVSSIYGSNEAFRN----------YGSGQLSEFEEESKEEQEMEKEEKL
P. yoelii       SVLLDNILTFNKNRKGKMSANARRKKIELALFNT-----IINDYSGNDSDGYLNVTNFSGALSSSYNEKPDGASSIYGSNEVFRN----------YGSGQLSEFEDENNEEQEMEKDETH
P. chabaudi     SVLLDNILIFNKNQKGKMSANARRKQIELALFN------TINDYSGNDSDDYLNITNFSGALSSSYNEKHDG-SSLYGSNEVFRN----------YGSGQLSEFGDENQEDQEMQKDEEL
P. knowlesi     SVLLDKILHVNH-KSAYPCEFSGASSSV-----------------YNDSDDYVKQLGEGGSYGYQDR----ADNLFSGSND----------------VSGSLGESDS-------HDAQEFL
P. vivax        SVLLDKILQVNN-KGAYPNEFSRASSSV-----------------YNESDEYVKQLGEGGSYGYQER----ADNLFGGSNE----------------VSGALAESDS-------LNAQETI
T. annulata     NQLILLHSKSTEGTKVEVKGARSEEVKEVTKSES-------------------------------------------------------------------------------TKDSEDTV
B. bovis        EALL--------------------------------------------------------------------------------------------------------------------
N. caninum      DTLLK-------------------------------------------------------------------------------------------------------------------
C. muris        EILLQIARGES-KDSTESNDNLGSDSPE-----------------------------------------------------------------------------------YEN----
C. parvum       ETLLMISKLELLKNEGIEIDGQGSEFMK---------------------------------------------------------------------------------------NLDSMI
C. hominis      ETLLMISKLELLKNEGIDIDGRGAEFMK---------------------------------------------------------------------------------------NLDSMI
T. thermophila  EVLINNI-----------------------------------------------------------------------------------------------------------------
S. pombe        KVLVEDI-----------------------------------------------------------------------------------------------------------------
S. cerevisiae   KVLIEDI-----------------------------------------------------------------------------------------------------------------
C. merolae      SVLIDQK-----------------------------------------------------------------------------------------------------------------
D. discoideum   DVLITHI-----------------------------------------------------------------------------------------------------------------
A. thaliana     NVLLDNV-----------------------------------------------------------------------------------------------------------------
D. melanogaster QVLIDQV-----------------------------------------------------------------------------------------------------------------
H. sapiens      QVLIEHI-----------------------------------------------------------------------------------------------------------------
```

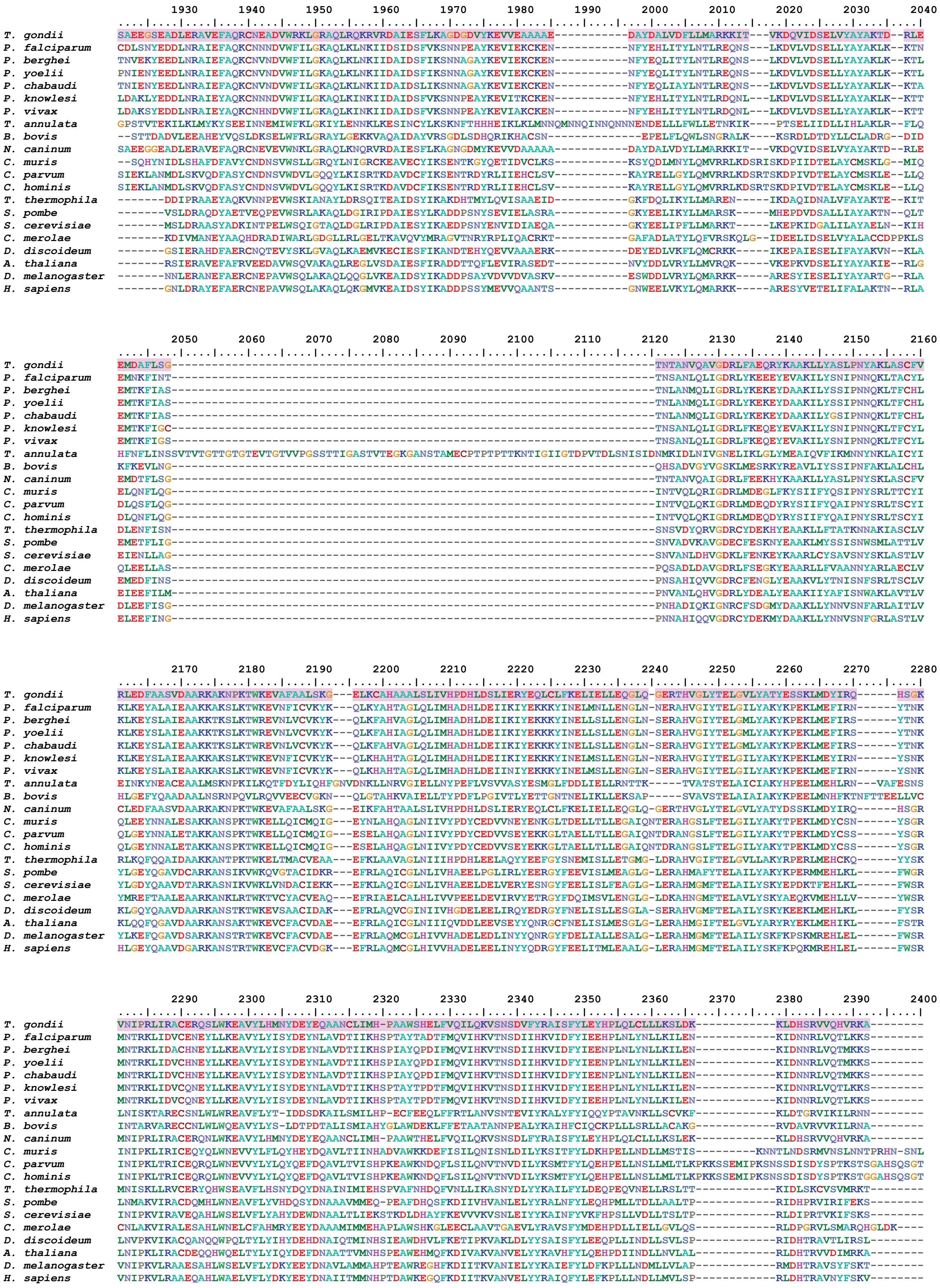

```
                      2410      2420      2430      2440      2450      2460      2470      2480      2490      2500      2510      2520
                 ....|....|....|....|....|....|....|....|....|....|....|....|....|....|....|....|....|....|....|....|....|....|....|....|
T. gondii        ----------------------------------------------------------GHLAVVEKYLRETQHLNITAVNEAVNELLVEGEDVDGLRESILEYDNFDQLALAQTLENHPR
P. falciparum    ----------------------------------------------------------NNLPLIQKYLEDIQAQNITAVNETLNEIYLQNDDYISLRNSIDEYDNFNQTNLLNKLENHKL
P. berghei       ----------------------------------------------------------NNLPLIQKYLEDIQTQNITSVNETLNEIYLENDDYISLRKSIDEYDNFNQTNLINKLENHKL
P. yoelii        ----------------------------------------------------------NNLPLIQKYLEDIQTQNITSVNETLNEIYLENDDYISLRKSIDEYDNFNQTNLINKLENHKL
P. chabaudi      ----------------------------------------------------------NNLPLIQKYLEDIQTQNITSVNETLNEIYLENDDYISLRKSIDEYDNFNQTNLINKLENHKL
P. knowlesi      ----------------------------------------------------------NNLPLIQKYLEDIQGQNITAVNETLNEIYLQNDDYISLRNSIDEYDNFNQTNLINKLENHKL
P. vivax         ----------------------------------------------------------NNLPLIQKYLEDIQGQNITAVNETLNEIYLQNDDYISLRNSIDDYDNFNQTNLINKLENHKL
T. annulata      ----------------------------------------------------------NCLQLAKEYFQQ-SDRNSQQINDTLYEIYVEEHDFESLEQDVSKYINYDYMKLCTLLEEHPL
B. bovis         ----------------------------------------------------------GCLGLARNYLEQVADKTSGAVNDALFEIYVEEEECELLERSLEKLTTFDQAKLCAMLQEHRL
N. caninum       ----------------------------------------------------------GHLAVVEKYLHDTQHLNITAVNEAVNELLVEGEDVDGLRESILEYDNFDQLALAQTLENHPR
C. muris         GSVNMGTNLADTP----QNTSNSLYNSSSINSRFDISSLCTRLIQQYFRKTGTATDSVASVALIQEFLESVCSENILAVNEALIDLYLNTKNVDKLQKLIMECDNYNQSNLSRKLESHPL
C. parvum        GRSGSNFDLANITTRFIQQYFSQGLSSTSSNTVGPITGISPRPSVG--KESGTQSGSNYDISIIQEFLESISDENIQVVNEALINLYIEKVEVEKLMKLILTCDNYDQAKLSARLEKHPM
C. hominis       GRTGSNFDLANITTRFIQQYFSQGLSSTSPNTVGPITGISPRPSVG--KESGSQSGSSYDISIIQEFLESISDENIQVVNEALINLYIEKVEVEKLMKLILTCDNYDQAKLSARLEKHPM
T. thermophila   ----------------------------------------------------------GYIALITPFLKSVQNANNKEVNEILNEIYLESFDYENLRQSITTYENFDQLTLAQNCERHTL
S. pombe         ----------------------------------------------------------ENTPLILNFMVAIQHLNIQAVNHAYNDLLIEMEDYQSLQDSIENYDHFDAIALARRLEKHSL
S. cerevisiae    ----------------------------------------------------------DNLPLIKPFLINVLPKNNSVVNQAYHDLMIEEEDYKALQDAVDSYDKFDQLGLASRLESHKL
C. merolae       ------------------------------------------------------FGPLGHVPLLKPYMLRVQDKNLAEVNEALNEVLIMEEDVDALVLSVERYPNFDQMALAQRLEKHEL
D. discoideum    ----------------------------------------------------------GHLPLVKPYLVSAQDQNVAALNEALNELYVEEEDYESLRSSIDANSNFGTIALAQKLEKHEL
A. thaliana      ----------------------------------------------------------GHLRLIKPYMVAVQSNNVSAVNEALNEIYAEEEDYDRLRESIDLHDSFDQIGLAQKIEKHEL
D. melanogaster  ----------------------------------------------------------GYLPLVKPYLRSVQSLNNKAINEALNGLLIDEEDYQGLRNSIDGFDNFDNIALAQKLEKHEL
H. sapiens       ----------------------------------------------------------KQLPLVKPYLRSVQNHNNKSVNESLNNLFITEEDYQALRTSIDAYDNFDNISLAQRLEKHEL

                      2530      2540      2550      2560      2570      2580      2590      2600      2610      2620      2630      2640
                 ....|....|....|....|....|....|....|....|....|....|....|....|....|....|....|....|....|....|....|....|....|....|....|
T. gondii        VEMRRLAALLFKKNRKFKQAIELSKRDRQYQDAIDAARDSGNTQLVGDLLRFFLADLEDPAGFAACLYTCYPLVKPDVALELAWRHKCMDYCMPFLIQVVREVTTRVDALDKKEETREKE
P. falciparum    PEMRRIAALLYKKNKKYKEAINLSKKEKQYKDAIEIARVSKNNVYIEDLINYFIE-SKNKEAFCACLIVCYDLLKPDYILEIVWTSGFKDQAMLYFIQIISDYTNQIEHMKKQIEDMEKE
P. berghei       AEMRRIAALLYKKNKKFKEAINLSKKEGQIKDAIDIARVSKNHLYVEDLINYFIQ-IKNKEALCACLIACYDILKPDYVLEIIWLSGFKDHAMLYFIQIISDYTQQIETMKKQIEDIEKE
P. yoelii        AEMRRIAALLYKKNKKFKEAINLSKKEGQIKDAIDIARVSKNHLYVEDLINYFIQ-IKNKEALCACLIACYDILKPDYVLEIIWLSGFKDHAMLYFIQIISDYTQQIETMKKQIEDIEKE
P. chabaudi      AEMRRIAALLYKKNKKFKEAINLSKKEGQIKDAIDIARVSKNHLYIEDLINYFIQ-IKNKEALCACLIACYDILKPDYVLEIIWLSGFKDQAMLYFIQIISDYTQQIETMKKQLEDIEKE
P. knowlesi      PEMRRIAALLYKKNKKYKEAINLSKKEEQYKDAIEIARVSKNNSYVEDLINFFIE-IKNKEALCACLIVCYDILKPDYILEIVWMSGYKDQAMLYFIQVISDYTNQIDVMKKQIEDMQKE
P. vivax         SEMRRIAALLYKKNKKYKEAINLSKKEEQYKDAIEIARVSKNNSYVEDLINFFIE-IKNKEALCACLIVCYDILKPDYILEIVWTSGFKDQAMLYFIQVISDYTSQIDAMKKQIEDMQKE
T. annulata      ARMREIGAKILSRHNHYSRASIIYMKNNNYIKAIDCARLSKSTQLVHETINNLIL-KEELNYLLVALVINFHLLDLPMVLESVWLNNVNLDVIMPLIIHLQRFINYKKPNSH--------
B. bovis         PKMRSIAAQLYIQSRDFGKAAVIYRRNGDYVDAIMGVQTSRSESLAMDMVRYFVD-QGLLEEFLVCLIVNYSLLDPADVLEYAWLHKVDMDILVPFLSHTIRTVSKAVSRPAAVQRPPPP
N. caninum       VEMRRLSALLFKKNRKFKQAIELSKRDRQYQDAIDAARDSGNSQLVGDLLRFFLSDLEDPAGFAACLYACYPLVKPDVALELAWRHKCMDYCMPFLIQVVREVTTRVDALDKKEETREKE
C. muris         NDFRRLAVKIFHKNADYQQALEICQKEMLIEDAIVVVYKSADTSLIENLLEFLLS-HKNIENFIACLYTCYEYLKPDVILELAWKYKCLDAAMPFFIQCVRDLTNKVENLEKRLDTVYLT
C. parvum        NDLRKLAVKILDKNSNYQQALSICQKEMLIDEAILVVYKSGNVALIEELLEFLLS-NNKKENFVACLYTCYEFLRPDTVMEMAWKHNCLDATMPFFIQSLRDMTNRIDVLEKKLETLQIT
C. hominis       NDLRKLAVKILDKNSNYQQALSICQKEMLIDEAILVVYKSGNVALIEELLEFLLS-NNKKENFVACLYTCYEFLRPDTVMEMAWKHNCLDATMPFFIQSLRDMTNRIDVLEKKLETLQIT
T. thermophila   LEFRRISAYLYRRIQKYEFSIALSKQDNIYRDAIETAQESGKVELVEDLLKFFVQ-KGEKEFITVTLYTCYELIRPDLAMEYAWRFNLFEFIMPYFIQITKELTSRVDTVQKKHEKREKE
S. pombe         LEFRRIAAYIYRKNKRWTQSIELSKQDRFYKDAIITARDSDQTTIAEDLMKYFVE-IGNYECFAAILYTCYHLLRNDLVMEISWRKGLQDYAYPYFINFQCEMFSKVLNLEKDLKDRQAV
S. cerevisiae    IFFKKIGALLYRRNKKWAKSLSILKEEKLWKDAIETAAISQDPKVVEALLTYFVE-TGNREGFVALLYAAYNLVRIEFVLEISWMNSLEDYIKPFEISIKKEQNDSIKKITEELAKKSGS
C. merolae       PLMRQVAVLLLRRSQKYERALELCKRDMAFREAAEIAAESGSASLAESIARYFLE-SNLKEAFTMALYLCYPLLRPDLVLEWTWRFGASDYAMPFYIQTLHDYGKRLEALERAERDHRTT
D. discoideum    LEFRRIAAYLYKKNNRWAQSVELSKKDKLYKDAIQSASDSKNPAIGEELLQYFVD-QQNNSAFAACLYTCYDFLKPDAVIELAWRNNILNYSFPYLIQYVKEYTTKVDQLVDDFKARQKK
A. thaliana      VEMRRVAAYIYKKAGRWKQSIALSKKDNMYKDCMETASQSGDHDLAEQLLVYFIE-QGKKECFATCLFVCYDLIRPDVALELAWINNMIDFAFPYLLQFIREYSGKVDELIKDKLEAQKE
D. melanogaster  TEFRRIAAYLYKGNNRWKQSVELCKKDKLYKDAMEYAAESCKQDIAEELLGWFLE-RDAYDCFAACLYQCYDLLRPDVILELAWKHKIVDFAMPYLIQVLREYTTKVDKLELNEAQR-EK
H. sapiens       IEFRRIAAYLFKGNNRWKQSVELCKKDSLYKDAMQYASESKDTELAEELLQWFLQ-EEKRECFGACLFTCYDLLRPDVVLETAWRHNIMDFAMPYFIQVMKEYLTKVDKLDASESLR-KE

                      2650      2660      2670      2680      2690      2700      2710      2720      2730      2740      2750
                 ....|....|....|....|....|....|....|....|....|....|....|....|....|....|....|....|....|....|....|....|....|
T. gondii        EEKQKNAPNDYVPDYT------------MPPTGSPLLPGMGGLALMPPSYTP-----------QSNAFPPTLGASGMMGSAP-ANLGQSFAPF-----------------
P. falciparum    KKMNKSAPNDYSAMN-----------------NQFNYSLNNNLSIMPP----------------QNNFMSSNS----FD---KYDMFNNNTHF-----------------
P. berghei       KKMNKSAPNDFSANT---------------ISNQFTYSLNKNLSIMPP----------------QNNYIPNNSD---FD---KYDMFNTNTHF-----------------
P. yoelii        KKMNKSAPNDYSANT---------------ISNQFTYSLNKNLSIMPP----------------QNNYIPNNSD---FD---KYDISYMTTCEFYFLCFFYFPYLFALRK
P. chabaudi      KKMNKSAPNDYSANT---------------ISNQFTYSLNKNLSIMPP----------------QNNYIPNNSD---FD---KYDMFNSNTHF-----------------
P. knowlesi      KKMNKSAPNDFAAN---------------SMPGQFNYSLNNHLSIMPP----------------QNTYMSSSSQYDKYD---KYDMFNNNSHF-----------------
P. vivax         KKMNKSAPNDFAANDFAANDFAGNDFAPNSMSGQFTYSLNNHLSIMPP----------------QNSYMSSSSQYDKFDKFDKYDMFNNNAHF-----------------
T. annulata      --------------------------------------------------------------------------------------------------------------
B. bovis         PQLSLPFH------------------------------------------------------------------------------------------------------
N. caninum       EEKQKSAPNDYVPDYN------------MPPTG-TMLPGMGGLALMPPSYTP-----------QANAFPPMGASAGMMGSAPPANLGQPFAPF-----------------
C. muris         DIAR---------NNASGTGG--------------NLHNGPSHNITEN-------------STNEIPALTN-HSYSDSKSGGLGNIGILGNLNKGGWV------------
C. parvum        DIATTASGGHPSVSNSQNISS--------------CVDMGPNSSISPG-------------LISSHHHVNNNNSNNNSNSNSSNTINNNYHLMSNNKLIFH---------
C. hominis       DIATTASGGHPSVSNSQNISS--------------CVDMGPNSSISPG-------------LISS-HHVNNNSSNNNSNSNSSNTINNNYHLMSNNKLIFH---------
T. thermophila   EEKKANQQLNQGMFMPTHDIL----------PT--ALMIGPPPGSMPGMNMPPMGGMPPMGGMPPMGGIPPMGGMPPMGGMPPMGGMGGFGTNPFGPQ------------
S. pombe         KSEEESASTIGAGIL------------------------GNTLMLTQG---------------PMANNNDQFDSFQQASPMPRL---GNF--------------------
S. cerevisiae    NEEHKD---------------------------------GQPLMLMNS---------------AMNVQPTGF--------------------------------------
C. merolae       NRKERTNAASGSESLLRSLTA----------STPMLMAGTPPTGPTPPSSLYFGA----SGPVPSMRMPSPAASAPAAAGMAGN---SARGRNPYFGT------------
D. discoideum    TEEEKEQQNIESSQYQPDLTN----------LSYGYAATGGMLALPPA---------------VGYQQQQQPQQMYNPNQMMGG---FQQNYNQYGGF------------
A. thaliana      VKAKEQEEKDVMSQQNMYAQL----------LP--LALPAPPMPGMGG---------------GGYGPPPQMGGMPGMSGMPPM---PPYGMPPMGGY------------
D. melanogaster  EDDSTEHKNIIQMEPQLMIT------------------AGPAMGIPPQ---------------YAQNYPPGAATVTAAGGRNMG---YPYL-------------------
H. sapiens       EEQATETQPIVYGQPQLMLT------------------AGPSVAVPPQ---------------APFGYG---YTAPPYGQPQPG---FGYSM------------------
```
