# Supplementary figures and images for: The Role of Clathrin in Post-Golgi Trafficking in Toxoplasma gondii
Source: PLoS One. 2013 Oct 11;8(10):e77620. doi: 10.1371/journal.pone.0077620 (PMC3795686; doi:10.1371/journal.pone.0077620)

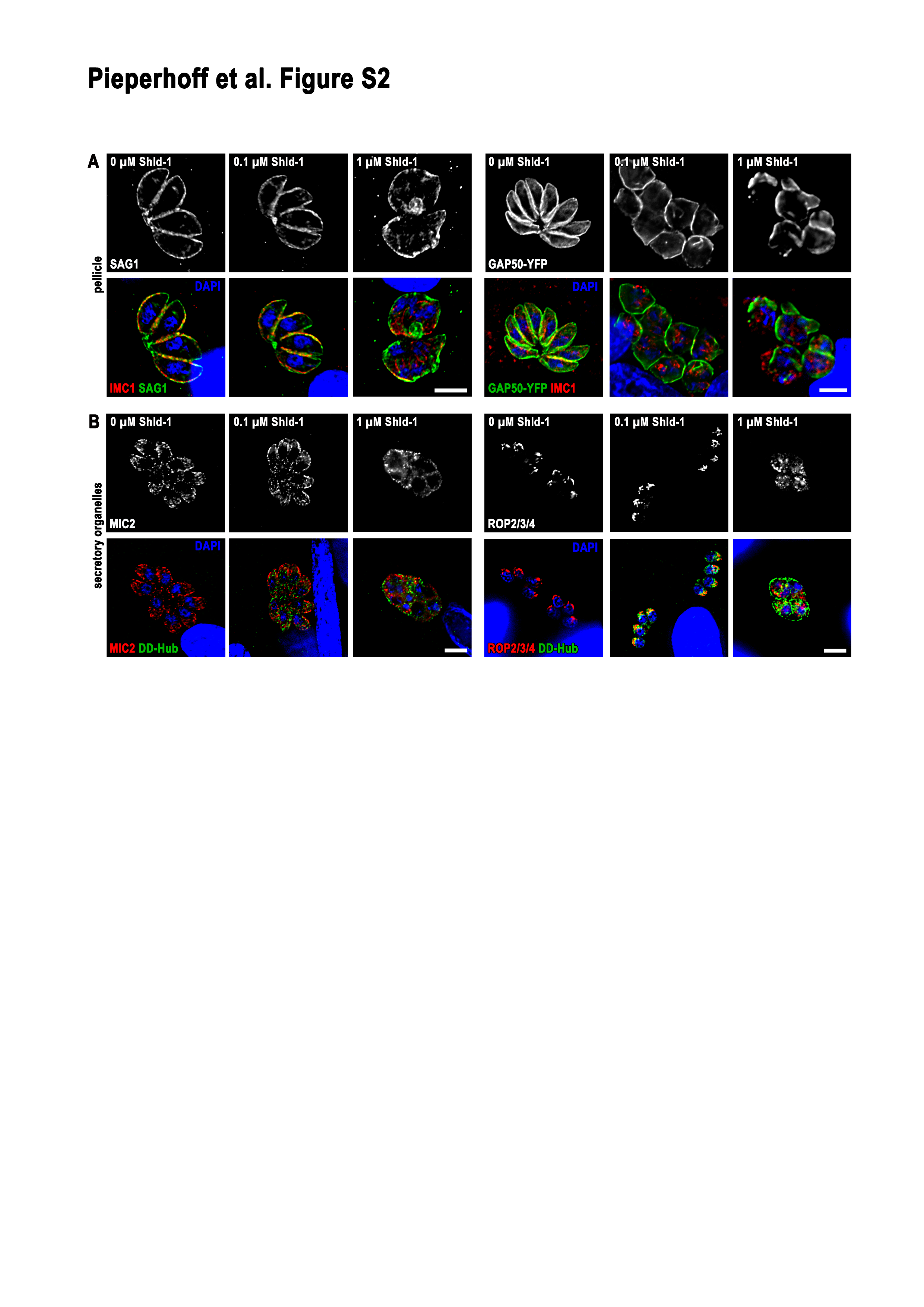

Supplement: Figure S2 — Immunofluorescence analysis of DD-Hub expressing parasites. Parasites were cultured for 24 hr in presence of indicated Shield-1 concentrations and analysed with indicated antibodies. (A) Colocalisations of IMC1 andSAG1 and GAP50-YFP and IMC1 respectively. (B) Colocalisations of DD-Hub and MIC2 and ROP2/3/4 respectively. Scale bars represent 10 μm. Immunoflourescence images are representative of at least three independent experiments and depicted abnormalities have been observed in 100% of 200 random examined vacuoles compaired to controls. (TIF) [file pone.0077620.s002.tif]

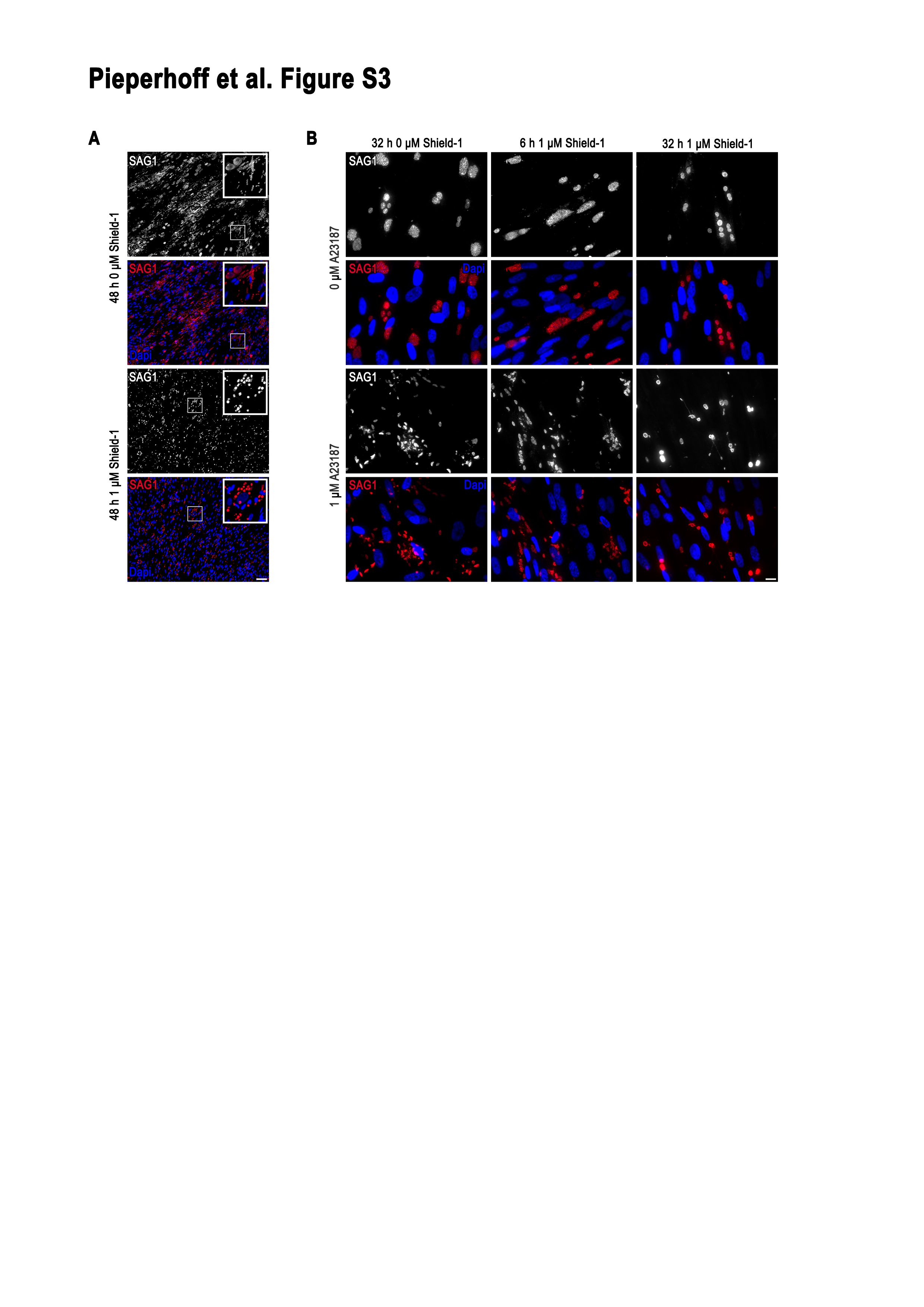

Supplement: Figure S3 — Natural but not induced egress is affected upon DD-Hub expression. (A) Natural egress. Immunofluorescence analysis of DD-Hub expressing parasites cultured for 48 hr in absence and presence of 1 μM Shield-1 and labelled with anti-SAG1-antibody. Inlets show threefold enlargements of the indicated area. Scale bar represents 100 μm. (B) Induced egress. Immunofluorescence analysis of DD-Hub expressing parasites grown for 32 hr and labelled with anti-SAG1-antibody. Treatment with 1 μM Shield-1 was either continuously or 6 hr prior to induction of host cell egress with 2 μM calcium ionophore A23187. Scale bar represents 25 μm. Whereas 6 hr incubation with Shield-1 has no influence on egress, long time exposure to Shield-1 results in an egress phenotype. Indicating that functional CHC1 might not play a role in RME and that the egress phenotype is due to the dramatic morphology changes of the parasites (see Figure 3 and 4, and S3). Immunoflourescence images are representative of at least three independent experiments. (TIF) [file pone.0077620.s003.tif]
