# Supplementary material for: The Role of Clathrin in Post-Golgi Trafficking in Toxoplasma gondii
Source: PLoS One. 2013 Oct 11;8(10):e77620. doi: 10.1371/journal.pone.0077620 (PMC3795686; doi:10.1371/journal.pone.0077620)
Supplement: Table S2 — Oligos used for cloning of T. gondii expression constructs and confirmation of specific construct integration and site specific recombination. (DOCX) [file pone.0077620.s005.docx]

**Table S2. Oligos used for cloning of *T. gondii* expression constructs and confirmation of specific construct integration and site specific recombination.**

| **Oligo** | **5’ - 3’ sequence** |
| --- | --- |
| CHC1-LIC-sense | TACTTCCAATCCAATTTAATGCGAAGCTCGCCATCTCGATGGGTGGAGAC |
| CHC1-LIC-antisense | TCCTCCACTTCCAATTTTAGCAAAGGGAGCGAAAGACTGTCCGAGATTGGC |
| CHC1-integration-sense | CGACTCGGGCAACACCCAAC |
| CHC1-integration-antisense | CAAGCCACAGCGGAACAA |
| Site-specific-recombination-sense | CGATGTTCCAGATTACGCTGCTGAC |
| Site-specific-recombination-antisense | CGAGATCCAATCCAATGCGGCCGC |
| Hub-sense | CCCCCTAGGAGGTTCGGACTGTTCGGGGAGGCTG |
| Hub-antisense | CCCTTAATTAAAAGGGAGCGAAAGACTGTCCGAG |
